# Refeeding-associated AMPK_γ1_ complex activity is a hallmark of health and longevity

**DOI:** 10.1038/s43587-023-00521-y

**Published:** 2023-11-13

**Authors:** Roberto Ripa, Eugen Ballhysa, Joachim D. Steiner, Raymond Laboy, Andrea Annibal, Nadine Hochhard, Christian Latza, Luca Dolfi, Chiara Calabrese, Anna M. Meyer, Maria Cristina Polidori, Roman-Ulrich Müller, Adam Antebi

**Affiliations:** 1 Max Planck Institute for Biology of Ageing, Cologne, Germany; 2 Cologne Graduate School for Ageing Research (CGA), Cologne, Germany; 3 Department II of Internal Medicine and Center for Molecular Medicine Cologne, University of Cologne, Faculty of Medicine and University Hospital Cologne, Cologne, Germany; 4 Cologne Excellence Cluster on Cellular Stress Responses in Aging-Associated Diseases (CECAD), University of Cologne, Cologne, Germany

**Keywords:** Ageing, Transcriptomics, Predictive markers

## Abstract

Late-life-initiated dietary interventions show limited efficacy in extending longevity or mitigating frailty, yet the underlying causes remain unclear. Here we studied the age-related fasting response of the short-lived killifish *Nothobranchius* *furzeri*. Transcriptomic analysis uncovered the existence of a fasting-like transcriptional program in the adipose tissue of old fish that overrides the feeding response, setting the tissue in persistent metabolic quiescence. The fasting–refeeding cycle triggers an inverse oscillatory expression of genes encoding the AMP-activated protein kinase (AMPK) regulatory subunits *Prkag1* (γ1) and *Prkag2* (γ2) in young individuals. Aging blunts such regulation, resulting in reduced *Prkag1* expression. Transgenic fish with sustained AMPK_γ1_ countered the fasting-like transcriptional program, exhibiting a more youthful feeding and fasting response in older age, improved metabolic health and longevity. Accordingly, *Prkag1* expression declines with age in human tissues and is associated with multimorbidity and multidimensional frailty risk. Thus, selective activation of AMPK_γ1_ prevents metabolic quiescence and preserves healthy aging in vertebrates, offering potential avenues for intervention.

## Abstract (web summary)

Age impacts the effect of dietary health and longevity interventions but the underlying mechanisms are incompletely understood. Here the authors study fasting in killifish and find that older animals exhibit a metabolic shift resembling a fasting-like program, which is counteracted by boosting the activity of AMPKγ1, promoting health and longevity.

## Main

Dietary interventions (DIs) that result in the periodic reduction or removal of caloric intake or specific diet components robustly promote health and longevity^1–4^ but impose a lifelong regimen that is not feasible in humans. To minimize such a burden, the question arises whether DI benefits can be induced at later time points; however, DIs initiated at old age in model organisms often fail to extend lifespan^5^ or can even lead to frailty^6–8^, indicating that such interventions are effective up to mid-life but may become detrimental later, yet the reasons remain unclear. As DIs entail cycles of fasting and refeeding^9^, great attention has been given to unraveling the physiological and molecular responses to nutrient removal and reintroduction. Yet whether the physiological response to fasting or refeeding becomes impaired in older animals remains unknown.

AMPK is an essential energy sensor that maintains energy homeostasis^10^. AMPK activation was previously shown to mediate lifespan extension and the beneficial effects of dietary restriction in invertebrate model organism^11–13^; however, its direct effect in promoting healthy aging and longevity in vertebrate species remains controversial^14,15^.

In this Article, we employed the short-lived turquoise killifish *Nothobranchius* *furzeri*, which lives 6–7 months and shows a systemic functional decline remarkably similar to mammals^16–18^, to study the age-related changes in response to food deprivation. By transcriptomic analysis, we observed that adipose tissue of older animals exhibited a fasting-like transcriptional program (FLTP) irrespective of their nutritional status (fed or fasted), characterized by widespread suppression of energy metabolism. At the gene level, we found that the regulatory AMPK γ-subunit *Prkag1* (γ1) expression was repressed by fasting and induced by refeeding. Such regulation was lost during aging resulting in chronic suppression of *Prkag1*. Mechanistically, we discovered that genetic activation of AMPK_γ1_ complex activity maintained a youthful response to feeding stimuli in the adipose tissue of older animals, thus sustaining tissue homeostasis late in life and ultimately promoting metabolic health and longevity. Notably, we also found that human *PRKAG1* expression declines as a function of age and is prognostic of multimorbidity and multidimensional frailty risk, suggesting that it is a critical causal biomarker of health. Thus, our results suggest that selective activation of the AMPK_γ1_ complex sustains tissue homeostasis late in life and promotes longevity.

## Results

### Aging initiates a fasting-like program in killifish adipose

**Fig. 1:**
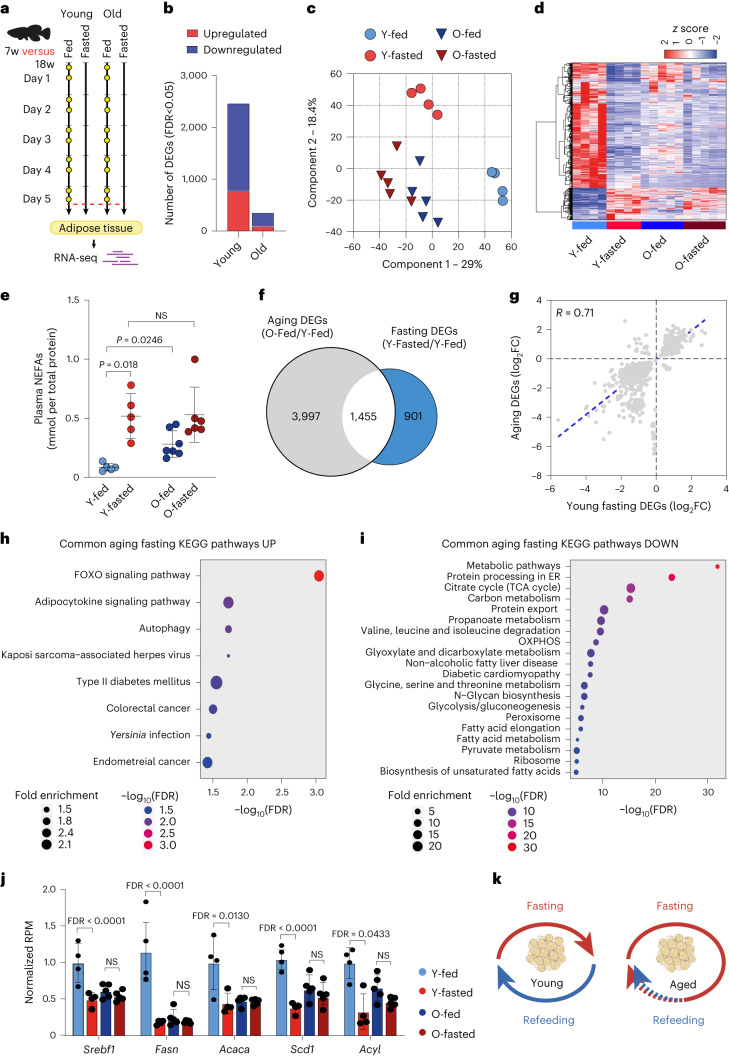
Aging alters the physiological response to fasting in the adipose tissue. **a**, Schematic of the food deprivation protocol in young (7 weeks) and old (18 weeks) fish. Control fish were fed twice daily (∼8:30 and ∼13:30, yellow dots) and killed 2 h after the last meal (red dashed line). Food-deprived fish were fasted for 5 d and killed along with control fish. **b**, Number of fasting-responsive genes in 7-week-old and 18-week-old fish (FDR < 0.05). **c**, PCA of log_2_-transformed and scaled gene expression data. **d**, Hierarchical clustering of expression changes for fasting-induced genes (FDR < 0.05). Colors represent the *z* score range. **e**, NEFAs quantification (mmol/plasma total protein quantification), Y-fed, *n* = 5; Y-fasted, *n* = 5; O-fed, *n* = 7; O-fasted, *n* = 6. Y, young; O, old. NS, not significant. **f**, Venn diagram showing the overlap between fasting and aging DEGs (hypergeometric test, two-tailed *P* = 4.5 × 10^−14^). **g**, Scatter-plot of log_2_ fold change (FC) for genes differentially expressed during aging and fasting (Pearson correlation, *r* = 0.71, two-tailed *P* < 0.0001). The aging effect is depicted on the *y* axis and the fasting effect on the *x* axis. **h**,**i**, KEGG pathway enrichment analysis of commonly up- (**h**) and downregulated genes (**I**) with FDR values displayed on a negative log_10_ scale along the *x* axis. **j**, DNL genes RNA-seq normalized counts in young fed (*n* = 4), young fasted (*n* = 4), old fed (*n* = 5), old fasted (*n* = 5).RPM, reads per milion. **k**, Schematic model showing the age-related changes in the killifish adipose tissue. Data in **e** and **j** are presented as mean ± s.d. Significance was measured by a two-way analysis of variance (ANOVA) (**e**) and a two-sided Wald test, adjusted for multiple testing (**j**). Source data

**Extended Data Fig. 1:**
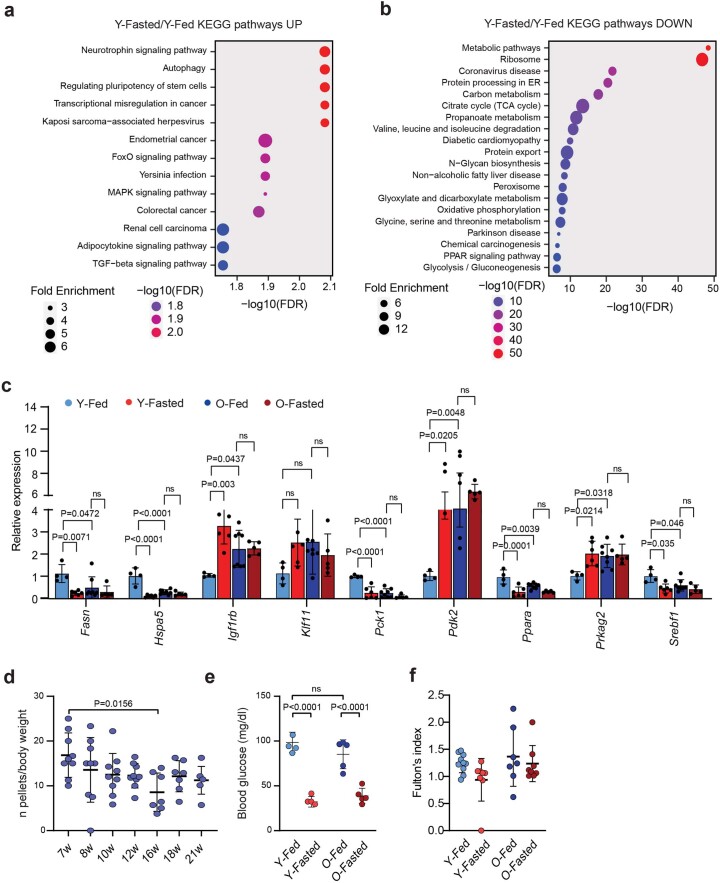
Aging is associated with a fasting-like transcriptional program (FLTP). **a, b**) KEGG pathway enrichment analysis of up- (**a**) and down-regulated genes (**b**) in young fasted/fed fish with FDR values displayed on a negative log10 scale along the x-axis. **c**) qPCR analysis of top fasting-regulated genes in young and old fish, young fed (n = 4), young fasted (n = 5), Old fed (n = 8), Old fasted (n = 5). Data values indicate fold change over the average value of young samples. **d**) Food intake analysis performed at different age points. 7 weeks old n = 9, 8 weeks old n = 8, 10 weeks old n = 9, 12 weeks old n = 9, 16 weeks old n = 7, 18 weeks old n = 7, 21 weeks old n = 5. **e**) Blood glucose measurements of young and old fish under feeding or fasting regime, Y-fed n = 4, Y-fasted n = 4, O-fed n = 5, O-fasted n = 5. **f**) Fulton’s index analysis in young and old killifish either fed or fasted. Y-fed n = 10, Y-fasted n = 8, O-fed n = 7, O-fasted n = 8. Data in **c-f** are presented as mean ± S.D. Significance was obtained by two-way ANOVA followed by Sidak multiple comparison test in **c, e**, and **f**, by one-way ANOVA followed by Tukey’s post hoc test in **d**. Source data

Using the turquoise killifish, we initially investigated how aging influences the physiological response to food deprivation. We primarily focused on the adipose tissue as it undergoes substantial remodeling under DIs and during aging^19^. As fish are ectotherms and kept at 27.5 °C, the adaptive response to food deprivation takes longer to achieve compared to mammals^20^. We observed that blood glucose levels off completely after 5 d of fasting, potentially corresponding to the near depletion of liver glycogen storage. Thus, young (7 weeks) and old (18 weeks) adult male killifish were fasted for 5 d, while age-matched control male fish were normally fed twice a day and killed 2 h after their last meal, together with fasted fish (Fig. 1a). We then performed transcriptome analysis on the visceral adipose tissue, comparing the fasted and fed conditions of young adult (Y-fasted/Y-fed) and old (O-fasted/O-fed) wild-type fish. We identified 2,469 differentially expressed genes (DEGs) in young fasted relative to young fed fish (false discovery rate (FDR) < 0.05; Fig. 1b). The majority of these DEGs were downregulated. KEGG enrichment analysis revealed downregulation of several metabolic pathways, including ribosome, tricarboxylic acid (TCA) cycle, oxidative phosphorylation (OXPHOS), glycolysis and fatty acid synthesis metabolism, among others (Extended Data Fig. 1a,b), reflecting a reduction in protein synthesis, metabolic rate and lipogenesis. Concomitantly, fasting induced the upregulation of autophagy and negative regulators of cell proliferation, such as *Pax6*, *Apc2* and *Bmp4* (ref. ^21^; Extended Data Fig. 1a,b). Thus, in line with mammalian data^1^, the physiological response to food deprivation reduces energy expenditure, protein synthesis and cellular proliferation in killifish.

Notably, gene expression changes in response to food deprivation seemed reduced in old fish. In fact, we could identify only 359 DEGs in old fasted relative to old fed fish (Fig. 1b). Accordingly, principal-component analysis (PCA) separated samples according to age (component 1). Still, only young groups according to diet (component 2) (Fig. 1c), suggesting that the transcriptional response to fasting is perturbed in old animals. To analyze the transcriptional similarity between old fed and fasted groups, we focused on up- or downregulated genes under fasting in young groups (Y-fasted/Y-fed) and evaluated their scaled expression across all groups. Of note, unsupervised hierarchical clustering analysis revealed that old fed fish adopted a transcriptional profile comparable with fasted fish (Fig. 1d).

We further validated the expression of some of the top fasting-responsive genes using an independent cohort of fish by qPCR analysis and observed the same pattern (Extended Data Fig. 1c). Fasting liberates non-esterified fatty acids (NEFAs) as an alternative energy substrate. Consistent with the transcriptomic data, older killifish exhibited elevated plasma NEFAs whether fed or fasted (Fig. 1e). Taken together, these data contravened our initial hypothesis of older animals being refractory to fasting, revealing instead that the transcriptional response to feeding is occluded by a persistent FLTP.

Aging can perturb feeding behavior. To determine whether FLTP simply arises from severe anorexia, we monitored the food intake across different age points (Methods and Extended Data Fig. 1d). Food intake progressively declined with age; however, none of the fish monitored at the later stages of life (18–21 weeks) showed signs of complete food deprivation. Consistently, post-prandial blood glucose levels were comparable between young and old fed fish (Extended Data Fig. 1e). Furthermore, we determined Fulton’s body condition factor (*K*), which indicates changes in the weight–length relationship, potentially reflecting adequate food intake and tissue composition (Extended Data Fig. 1f). *K* values ≥ 1 indicate good growth conditions, whereas values ≤ 1 indicate poor growth conditions. In line with the data above, both young and old fish showed *K* ≥ 1, denoting a generally good growth condition even at 18 weeks of age.

To gain more insight into the nature of the age-related FLTP, we directly compared the transcriptional response of fasting (Y-fasted/Y-fed) with that of aging (O-fed/Y-fed). Notably, this comparison showed an overlap of 1,455 genes (22.9%, *P* = 4.3 × 10^−14^) (Fig. 1f). Furthermore, a large percentage (92%) of overlapping DEGs were altered in the same direction by fasting and aging (Fig. 1g), indicating similar transcriptional regulation. KEGG enrichment analysis of the overlapping DEGs revealed a strong suppression of energy metabolism-related pathways (Fig. 1h,i), as well as several lipid metabolism biosynthetic pathways. Notably, old fish showed increased expression of fasting-induced FOXO signaling. Consistently, FOXO expression was found to increase in the gut of old flies, contributing to the disruption of lipid homeostasis^22^. Furthermore, dietary-resistant old mice have reduced de novo lipogenesis (DNL) gene expression, crucial for the production of new adipocytes^5^. We observed the same pattern in old killifish, indicative of an evolutionary conserved regulation (Fig. 1j). Thus, the adipose feeding response is overrun by a persistent FLTP in old fish that suppresses energy and biosynthetic lipid metabolism (Fig. 1k).

**Fig. 2:**
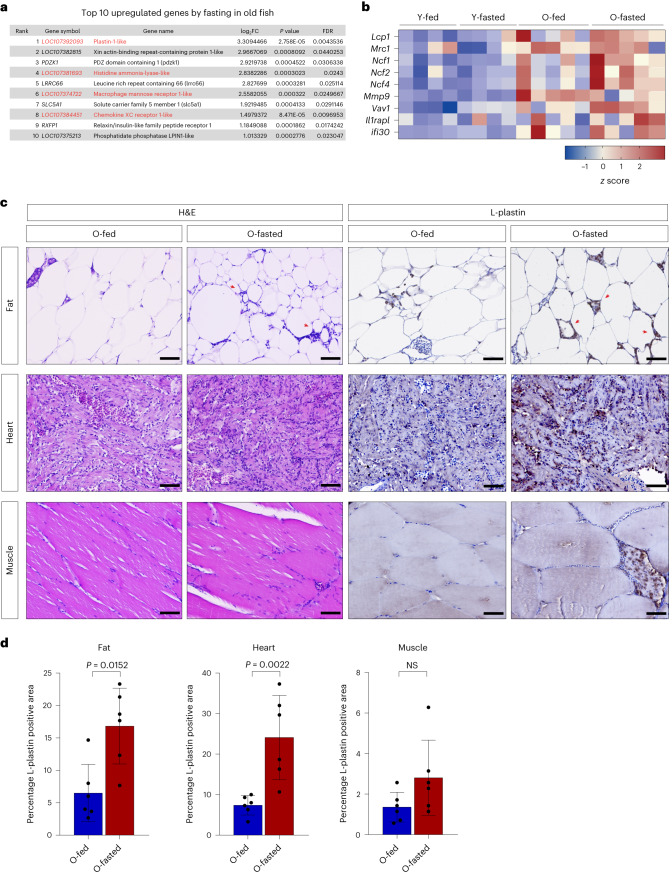
Fasting is associated with immune response in old animals. **a**, Top ten fasting-induced genes in old fasted compared to old fed fish. Immune-related genes are indicated in red. **b**, A *z* score heat map of immune response-associated genes across all the samples. **c**, Hematoxylin and eosin (H&E) staining (on the left) and L-plastin immunostaining (on the right). Red arrowheads indicate crown-like structures in the adipose tissue. **d**, Quantification of the area occupied by L-plastin-positive cells over the total analyzed area, *n* = 6 fish/group. Scale bar, 100 µm. Data in **d** are presented as mean ± s.d. Significance was measured by a two-tailed Mann–Whitney *U*-test. Source data

Of note, fasting induced the expression of several inflammatory genes in the adipose of old, but not young fish. In fact, despite the reduced number of fasting-responsive genes in old fish, we noticed that four out of the top ten fasting upregulated genes were implicated in the innate immune response (Fig. 2a) potentially indicating an ongoing inflammatory process. To assess whether this was an age-specific response, we evaluated the regulation of inflammatory genes across all samples. The expression of such genes was low in young animals whether fasted or not, higher in old fed animals and, notably, further increased in old fasted animals (Fig. 2b). Thus, the physiological response to food deprivation in the adipose tissue is associated with an enhanced inflammatory signature in old animals.

Consistently, standard histology revealed a high number of infiltrating cells in the adipose tissue, skeletal muscle and heart of old fasted animals (Fig. 2c). As such, we stained for L-plastin, a pan-leukocyte marker in fish, revealing that old fasted fish showed a higher presence of immunoreactive cells in such organs (Fig. 2c,d). Notably, L-plastin^+^ reactive cells mostly surrounded adipocytes (crown-like structures) or, in some cases, skeletal muscle fibers. Thus, our data indicate that late-life fasting can exacerbate a pre-existing age-related inflammatory process.

### FLTP is associated with dysregulated AMPK γ subunit expression

**Fig. 3:**
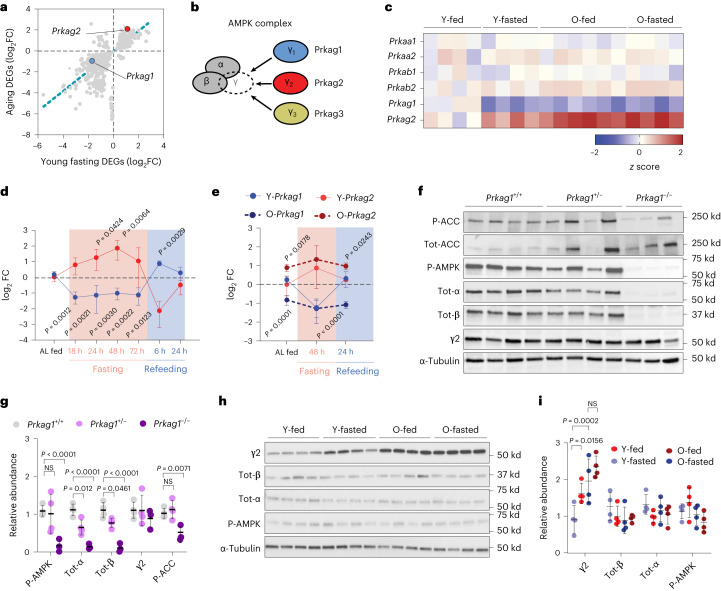
Fasting and aging modulate the expression of AMPK γ subunits. **a**, Scatter-plot of log_2_ FC for genes differentially expressed during aging and fasting. The blue and red dots indicate the expression of *Prkag1* and *Prkag2*, respectively, during aging and fasting. **b**, Schematic representation of the AMPK complex and the three different γ subunits. **c**, A *z* score heat map of AMPK subunits gene expression across all the samples. **d**, log_2_ FC of *Prkag1* and *Prkag2* relative expression upon fasting (0, 18, 24, 48 and 72 h) and refeeding (72 h of fasting followed by 6 and 24 h of refeeding) in 7-week-old fish, *n* = 4 fish per group. Data values indicate FC over the average value of ad libitum (AL) fed control animals. **e**, log_2_ fold change of *Prkag1* and *Prkag2* relative expression upon fasting (48 h) and refeeding (48 h of fasting followed by 24 h of refeeding) in young (7 weeks old, solid lines) and old individuals (18 weeks old, dashed lines), *n* = 4 per group. Data values indicate FC over the average value of young fed control animals (AL). **f**,**g**, Representative immunoblot and relative quantification showing the expression of γ2 subunit, total-α, total-β, phospho-Thr172-AMPKα, P-ACC, total-ACC and α-tubulin in wild-type (*n* = 4), *Prkag1*^+/−^ (*n* = 4), and *Prkag1*^−/−^ (*n* = 3) adipose tissue. **h**,**i**, Representative immunoblot and relative quantification of γ2 subunit, total-α, total-β, phospho-Thr172-AMPKα and α-tubulin in fasted and old fish adipose tissue. Quantification of protein abundance by densitometric analysis, *n* = 4 fish per group; each dot represents an independent biological replicate. Data are presented as mean ± s.d. (**d**,**e**,**g**,**i**). Significance was obtained by a one-way ANOVA followed by Tukey’s post hoc test (**d**,**g**) and a two-way ANOVA followed by Sidak multiple comparison test (**e**,**i**). Source data

**Extended Data Fig. 2:**
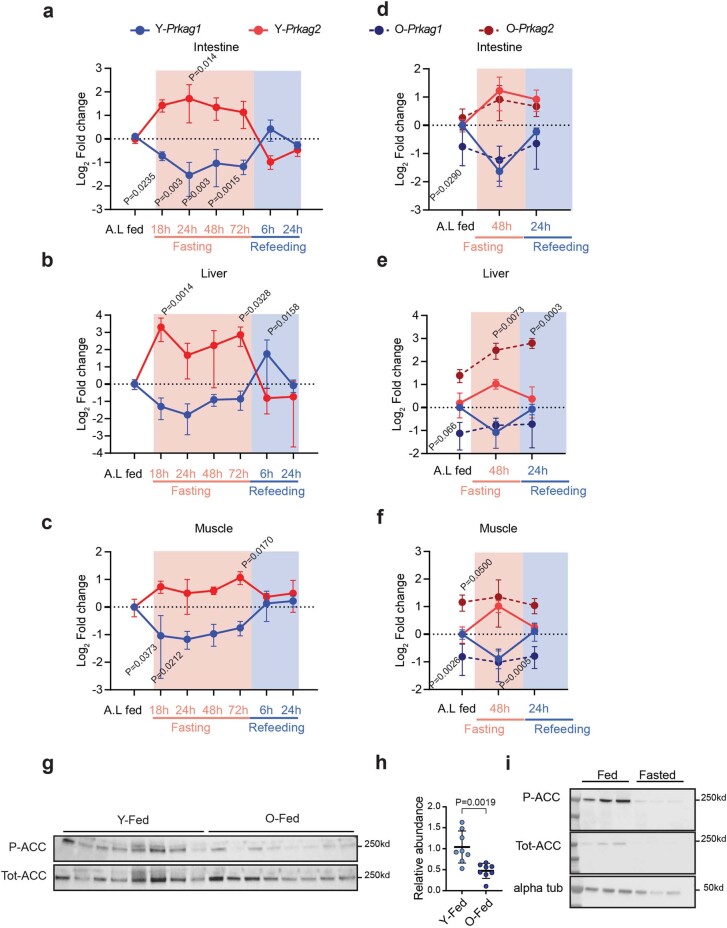
Nutritional-dependent regulation of AMPK γ subunits mRNAs in different tissues. **a–c**) Log2 fold change of *Prkag1* and *Prkag2* relative expression upon fasting (0, 18, 24, 48, and 72 h) and refeeding (72 h of fasting followed by 6 and 24 h of refeeding) in the intestine, muscle, and liver in 7 weeks old fish, n = 4 fish/group. Data values indicate fold change over the average value of *ad libitum* fed control animals (A.L.). **d–f**) Log2 fold change of *Prkag1* and *Prkag2* relative expression upon fasting (48 h) and refeeding (48 h of fasting followed by 24 h of refeeding) in young (7 weeks old, solid lines) and old individuals (18 weeks old, dashed lines), n = 4 fish/group. Data values indicate fold change over the average value of young fed control animals (A.L). **g–h**) Representative immunoblot and relative quantification showing the expression of P-ACC, total-ACC, in the adipose of 7 weeks (n = 8) and 18 weeks (n = 8) wild type fish. **i)** Representative immunoblot showing the expression of P-ACC, total-ACC in the adipose of 7 weeks under feeding (n = 3) and fasting conditions (n = 3). Data in **a**-**f** and **h** are presented as mean ± S.D. data. Significance was obtained by one-way ANOVA followed by Tukey’s post hoc test in **a**-**c**, two-way ANOVA followed by Sidak multiple comparison test in **d-f**, by two-tailed Mann-Whitney U test in **h**. Source data

The evidence that FLTP was primarily associated with the suppression of energy metabolism pointed at a possible dysregulated expression of nutrient-sensing or energy-related genes. Of note, we observed that the γ subunits of AMPK were markedly regulated in our FLTP data (Fig. 3a). AMPK is an evolutionary conserved fuel-sensing enzyme that sustains energy metabolism under different physiological conditions and comprises three different γ subunits (Fig. 3b). Yet, the physiological relevance of these different γ subunits remains unclear. Fasting induced a downregulation of the AMPK γ1 subunit encoding gene *Prkag1* and upregulation of the γ2 encoding gene *Prkag2* at the transcriptional level (Fig. 3c), suggesting a change in the stoichiometry of γ subunits in the transition from feeding to fasting; however, this pattern was not visible in fed or fasted old animals, suggesting a possible dysregulation of AMPK signaling. Notably, a nutritional-dependent modulation of the AMPK γ subunits was recently observed in humans undergoing a fasting regimen, hinting at an evolutionarily conserved process^23^. Furthermore, rare variants of specific AMPK γ subunits were identified to be associated with human longevity^24^. Thus, we wished to explore further the potential connection between nutritional state and AMPK γ subunits expression. As we had initially observed regulation of the γ subunits upon 5 d of food deprivation, we next sought to determine their expression upon shorter fasting regimes and refeeding. Five groups of young killifish were subjected to fasting for 0 to 72 h and two groups fasted for 72 h and subsequently re-fed for 6 or 24 h. *Prkag1* expression was downregulated within 18 h after food deprivation and seemed downregulated in all fasted groups, but rapidly increased within 6 h of refeeding to the level of ad libitum fed conditions (Fig. 3d). Conversely, *Prkag2* expression increased at 18 h of fasting, peaked at 48 h and rapidly decreased upon refeeding (Fig. 3d). Extending the analysis to other organs, we observed a similar expression pattern in skeletal muscle, liver and intestine (Extended Data Fig. 2a–c). Thus, our findings revealed that AMPK γ subunits exhibit an inverse oscillatory expression in response to the nutritional state. Next, we evaluated the effect of aging on γ subunit expression dynamics. In this case, we monitored the expression of γ subunits at 0 and 48 h of fasting and 48 h of fasting followed by 24 h of refeeding. Of note, the expression of γ subunits in most of the analyzed tissues was not restored to a steady state upon refeeding in old animals (Fig. 3e and Extended Data Fig. 2d–f). Thus, FLTP associates with a dysregulated expression of AMPK γ subunits in old animals.

**Extended Data Fig. 3:**
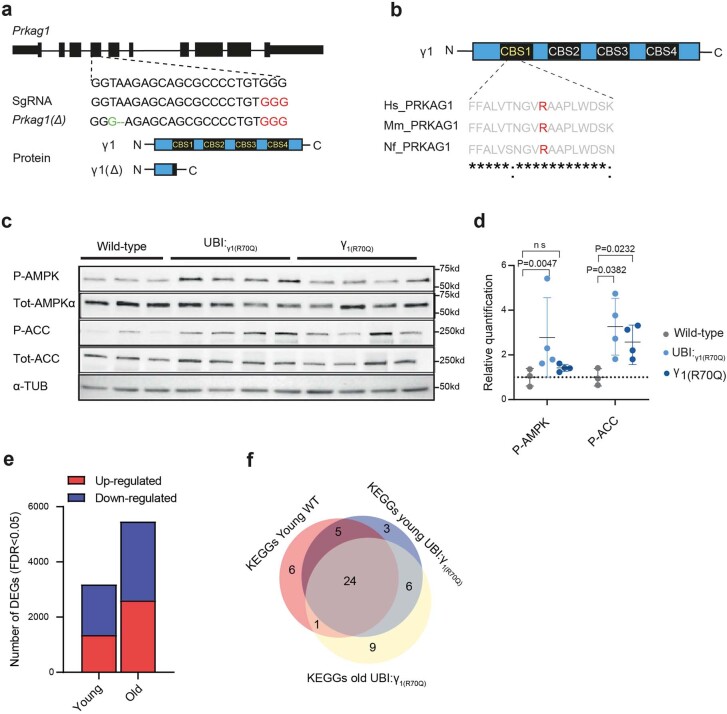
Sustained activation of the AMPKγ1 complex prevents FLTP. **a**) Generation of a CRISPR *Prkag1* mutant line. Location of the sgRNA targeting *Prkag1* exon 4 (Top). Green highlights the precise editing of DNA exon 4 sequence. Protospace adjacent motif (PAM) is indicated in red. **b**) Evolutionarily conserved AMPK γ1 amino acid sequence between humans, mice, and killifish. Red highlights the critical amino acid residue R70 (R68 in killifish). **c, d**) Representative Western blots and densitometric analysis of phospho-Thr172-AMPKα, total-AMPKα, phospho-S79-ACC, total-ACC, in 18 weeks old killifish adipose tissue under normal fed conditions. Old wild-type n = 3, UBI:γ_1(R70Q)_ n = 4, and γ1_(R70Q)_ n = 4. α-tubulin was used as a loading control. **e**) Fasting-responsive genes in young and old UBI:γ_1(R70Q)_ fish (FDR < 0.05) **f**) Venn diagram depicting the overlap of KEGG pathways differentially regulated by fasting in young wild-type, young Ubi:γ_1(R70Q)_, old Ubi:γ_1(R70Q)_ fish (hypergeometric test two tailed P = 1.2e-12). **h**) Data in **d** are presented as mean ± S.D. Significance was obtained by one-way ANOVA followed by Tukey’s post hoc test in **d**. Source data

Next, we tested whether the differential expression of γ subunits genes results in a subunit exchange within the AMPK complex during fasting and aging. Yeast studies showed that AMPK is an obligate heterotrimeric complex whose overall stability relies on the stoichiometric balance of α, β and γ subunits^25^. Consistently, we found that a γ1-deficient fish line (Extended Data Fig. 3a) caused a reduction of total α and β subunits, but not γ2 (Fig. 3f,g); however, we could not monitor γ1 protein levels because commercially available antibodies did not cross-react with the killifish protein. Thus, in line with previous mice data^26,27^, the removal of AMPK γ1 results in decreased levels of the other complex subunits but not changes in γ2 abundance. We also observed a reduction of acetyl-CoA carboxylase (ACC) phosphorylation level (Fig. 3f,g), indicating that AMPK_γ1_ is catalytically active and regulates energy metabolism even under normal feeding conditions. Consistently we also observed reduced P-ACC levels in old fed animals (Extended Data Fig. 2g,h), indicating that the age-related decline in *Prkag1* leads to reduced fatty acid oxidation. Next, we monitored the AMPK subunit remodeling upon fasting and aging. In accordance with our transcriptomic data, γ2 protein abundance was elevated during fasting in young fish compared to the fed controls. In contrast, it remained high independent of the diet regime in old fish; however, the levels of total α and β subunits remained constant (Fig. 3h,i), indicating, by inference, that the γ2 increase compensates for any γ1 reduction. Taken together, these data suggest that the total amount of γ subunits remains constant during fasting and aging, but the AMPK γ ratio changes in favor of γ2. Notably, despite a 5-d fasting period, there were no discernible changes in P-AMPKα levels (Fig. 3h,i). Nevertheless, the possibility remains that P-AMPK levels may experience a transient increase during an earlier phase of fasting, followed by a subsequent decline. We also sought to examine P-ACC levels; however, fasting triggered a complete degradation of ACC (Extended Data Fig. 2i), making the measurement of P-ACC levels unattainable under these circumstances.

In summary, our findings suggest that both fasting and aging induce a remodeling of the AMPK complex composition. Of note, this regulatory process seems to occur independently of alterations in AMPK-ser172 phosphorylation.

### AMPK_γ1_ activity prevents FLTP and preserves energy metabolism

Humans or mice carrying γ2 gain-of-function mutations exhibit cardiometabolic dysfunctions, insulin resistance and obesity^14,15^. Some of these alterations were also recapitulated by overexpressing the wild-type γ2 mRNA in mice^28^. Furthermore, γ2 expression was found to increase threefold in humans affected by Alzheimer’s disease (AD) and the protein level positively correlated with Aβ accumulation in the AD brain^29^. As, in killifish, higher levels of AMPK_γ2_ complex are associated with FLTP and metabolic quiescence, we asked whether selective stimulation of the refeeding-associated AMPK_γ1_ complex could revert such a condition.

**Fig. 4:**
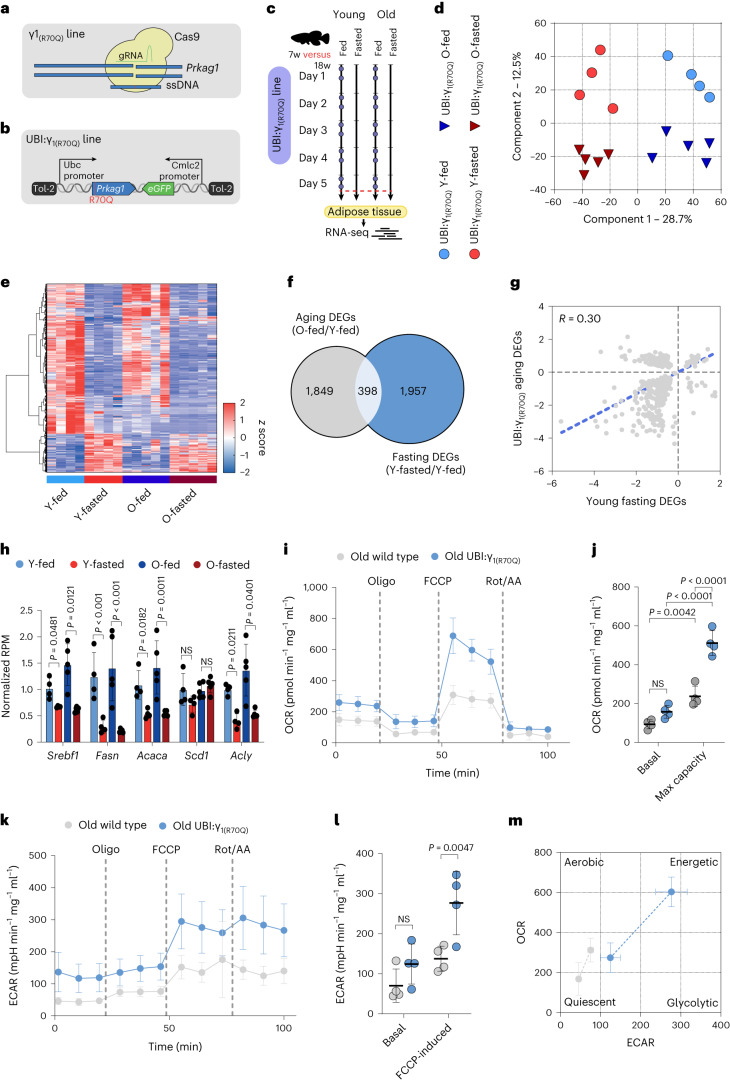
Sustained activation of AMPK_γ1_ prevents FLTP in old fish. **a**,**b**, Schematic illustration showing the CRISPR/Cas9 line generation (γ_1(R70Q)_) in **a**, and the Tol-2 line (UBI:γ_1(R70Q)_) in **b**. **c**, Schematic of the food deprivation protocol. Control fish were fed twice daily (~8:30 and ~13:30, blue dots) and killed 2 h after the last meal (red dashed line). Fasted fish were fasted for 5 d and killed along with control fish. **d**, PCA of log_2_-transformed and scaled gene expression data. **e**, Hierarchical clustering of expression changes for fasting-induced genes (FDR < 0.05). Colors represent the *z* score range. **f**, Venn diagram showing the overlap between fasting and aging DEGs (hypergeometric test, two-tailed *P* = 0.99). **g**, Scatter-plot of log_2_ fold changes for genes differentially expressed during aging and fasting (Pearson correlation, *r* = 0.30, two-tailed *P* < 0.001). The aging effect is depicted on the *y* axis, and the fasting effect on the *x* axis. **h**, DNL genes RNA-seq normalized counts in young UBI:γ_1(R70Q)_ fed (*n* = 4), young UBI:γ_1(R70Q)_ fasted (*n* = 4), old UBI:γ_1(R70Q)_ fed (*n* = 5) and old UBI:γ_1(R70Q)_ fasted (*n* = 5). **i**,**j**, OCR analysis and relative quantification of freshly isolated visceral adipose primary cells from 18 weeks old wild-type (*n* = 4) and UBI:γ_1(R70Q)_ (*n* = 4). The analysis was performed under basal conditions and in response to mitochondrial inhibitors (oligomycin (oligo), FCCP and rotenone (rot)/antimycin A (AA)). Values were normalized to total protein concentration. **k**,**l**, Glycolytic function profile and relative quantification by ECAR of freshly isolated visceral adipose primary cells from 18-week-old wild-type (*n* = 4) and UBI:γ_1(R70Q)_ (*n* = 4). The analysis was performed under basal conditions and in response to mitochondrial inhibitors (oligomycin, FCCP and rotenone/antimycin A). Values were normalized to total protein concentration. **m**, Energy phenotype profile showing basal and maximal OCR and ECAR of freshly isolated visceral adipose cells from 18-week-old wild-type (*n* = 4) and UBI:γ_1(R70Q)_ (*n* = 4). Data are presented as mean ± s.d. and significance was measured by a two-sided Wald test, adjusted for multiple testing (**h**) and by two-way ANOVA followed by Sidak test (**j**,**l**). Source data

The γ1 R70Q mutation uncouples the inhibitory properties of ATP and results in constitutive activation of AMPK_γ1_ complex^30^. Given the high conservation of AMPK subunits across killifish and mammals (Extended Data Fig. 3b), we introduced the R70Q mutation into the endogenous γ1 locus by CRISPR genome engineering (referred to as γ_1(R70Q)_, Fig. 4a) and, as γ1 expression is diminished during aging, we generated another line overexpressing constitutively expressed γ1 subunit bearing the R70Q mutation (referred to as UBI:γ_1(R70Q)_; Fig. 4b, Methods and Extended Data Fig. 8). We then determined the phosphorylation levels of the AMPK α subunit residue at Thr172, required for AMPK activation and its downstream substrate ACC in the adipose tissue of middle-aged wild-type, UBI:γ_1(R70Q)_ and γ_1(R70Q)_ fish. The UBI:γ_1(R70Q)_ line showed elevated P-AMPKα levels compared to the wild-type, whereas both mutant lines showed elevated P-ACC levels, with a stronger effect in the UBI:γ_1(R70Q)_ line (Extended Data Fig. 3c,d). Thus, in line with mammalian data, the introduction of the R70Q mutation resulted in chronic activation of the AMPK_γ1_ complex.

**Extended Data Fig. 4:**
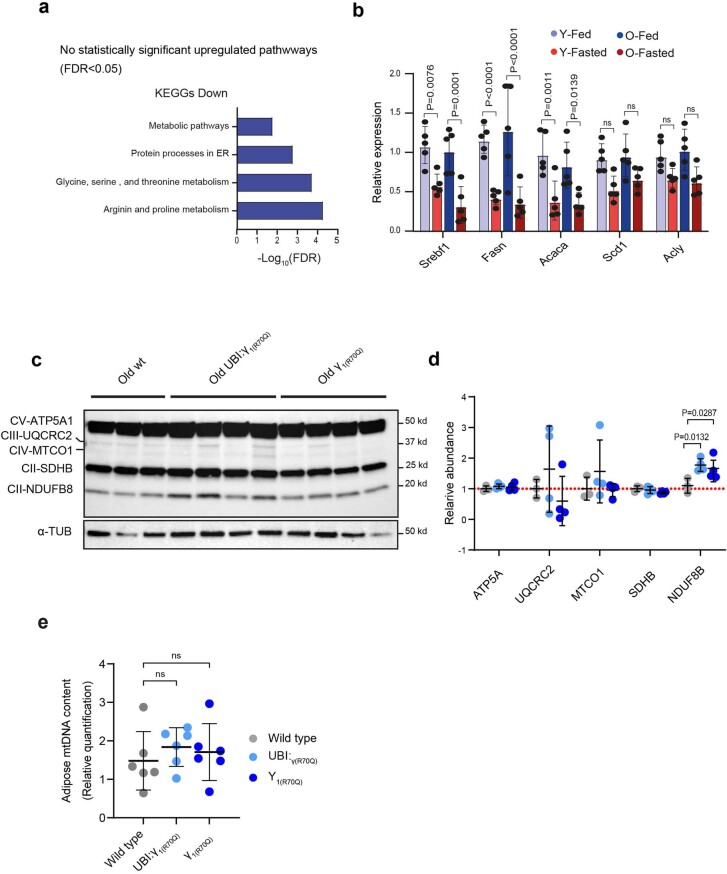
Sustained activation of AMPKγ1 sustain energy metabolism late in life. **a**) KEGG pathway enrichment analysis of differentially regulated genes comparing old to young fed UBI:γ_1(R70Q)_ fish with FDR values displayed on a negative log10 scale along the x-axis. **b**) qPCR analysis of DNL genes in young and old fed and fasted γ_1(R70Q)_ fish, n = 5 fish/group. **c, d**) Representative Western blots and densitometric analysis of the mitochondrial electron-transport chain components. Old wild-type n = 3, UBI:γ_1(R70Q)_ n = 4, and γ1_(R70Q)_ n = 4. **e**) mtDNA content in old wild-type (n = 6), UBI:γ_1(R70Q)_ (n = 6), and γ1_(R70Q)_ (n = 6) lines. Data in **b, d**, and **e** are presented as mean ± S.D. Significance was obtained by one-way ANOVA followed by Tukey’s post hoc test in **d-e**, by Two-way ANOVA followed by Sidak test in **b**. Source data

We next determined the physiological response to food deprivation in fish with increased AMPK_γ1_ activity, as we did previously with wild-type animals (Fig. 4c). For this experiment, we employed the UBI:γ_1(R70Q)_ line as it showed a more robust AMPK_γ1_ complex activation. In contrast to wild-type fish, PCA separated samples according to both age and diet regimen (Fig. 4d). Furthermore, the main source of variation (component 1) was diet regimen, whereas the second was age (component 2). In line with the PCA, differential expression analysis identified 3,185 DEGs comparing young-fasted/young-fed and, notably, 5,465 DEGs comparing old fasted/old fed (Extended Data Fig. 3e), indicating a strong transcriptional fasting response in old animals. Despite the high number of DEGs induced by fasting, KEGG pathway analysis revealed that the differentially regulated processes in UBI:γ_1(R70Q)_ were consistent with those observed in wild-type (Extended Data Fig. 3f). Furthermore, unlike wild-type animals, the scaled expression profile of fasting-responsive genes (FDR < 0.05) in old fed mutant fish more closely resembled the profile of fed fish (Fig. 4e). Aging and fasting transcriptional responses overlapped only 398 genes (9%) (Fig. 4f). Of those, 36% showed an opposite regulation (Fig. 4g). FLTP was primarily characterized by a marked reduction of energy and lipid metabolism; however, UBI:γ_1(R70Q)_ fish showed little or no sign of reduced energy and lipid metabolism pathways during aging (Extended Data Fig. 4a). In line with this, UBI:γ_1(R70Q)_ did not show age-related reduced expression of DNL genes under the feeding regimen (Fig. 4h). We further validated the DNL genes expression in γ_1(R70Q)_ fish (Extended Data Fig. 4b) and observed a similar pattern, indicating that these mutant lines share a similar transcriptional profile. Finally, to validate the energy metabolism transcriptomic data, we dissociated cells from old wild-type and UBI:γ_1(R70Q)_ adipose tissue and measured the oxygen consumption rate (OCR) and the extracellular acidification rate (ECAR) with a Seahorse Analyzer (Methods). UBI:γ_1(R70Q)_ cells showed a tendency to increase basal OCR, although this did not reach significance. We did, however, see a clear increase in maximal OCR upon FCCP addition, suggesting better mitochondrial fitness (Fig. 4i,j). UBI:γ_1(R70Q)_ cells also showed increased glycolytic function compared to wild-type (Fig. 4k,l). Altogether these data indicate that UBI:γ_1(R70Q)_ cells are more metabolically active compared to wild-type under basal conditions and have more metabolic potential under stress conditions in old animals (Fig. 4m). To assess mitochondria biogenesis we measured expression levels of OXPHOS components and mitochondrial DNA (mtDNA) content in the adipose of old wild-type and mutant lines; however, we only saw an increase of complex 1 subunit NDUFb8 (Extended Data Fig. 4c,d) in both mutant lines compared to wild-type and no significant changes in mtDNA content (Extended Data Fig. 4e), indicating no consistent increase in mitochondrial biogenesis. Thus, sustained AMPK_γ1_ complex activation counters the age-associated FLTP and maintains energy and lipid metabolism responsiveness to feeding late in life.

### AMPK_γ1_ activation maintains tissue homeostasis in late life

**Fig. 5:**
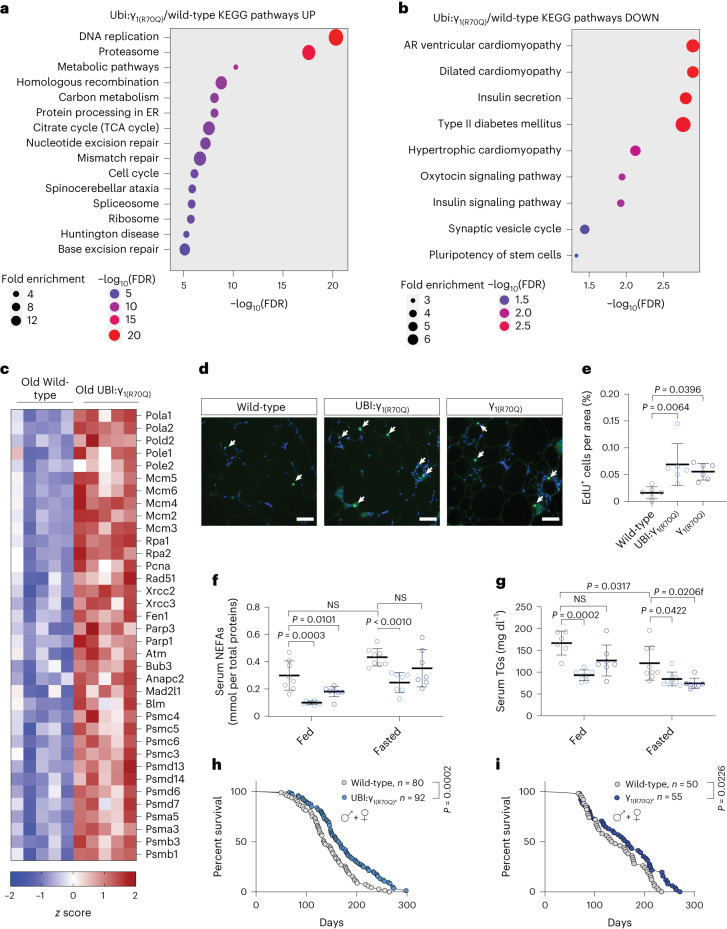
Sustained activation of the AMPK_γ1_ complex promotes metabolic health and longevity. **a**,**b**, KEGG pathway enrichment analysis of up- (**a**) and downregulated genes (**b**) in old UBI:γ_1(R70Q)_ compared to old wild-type fish under the normal fed condition. FDR values are represented by a negative log_10_ scale (*x* axis). **c**, A *z* score heat map showing DEGs involved in DNA synthesis and repair and proteostasis. **d**,**e**, Representative images and relative quantification of EdU-labeled cells in the adipose tissue of 18-week-old wild-type and mutant lines; 18-week-old wild-type (*n* = 5), UBI:γ_1(R70Q)_ (*n* = 6), γ_1(R70Q)_ (*n* = 6). Scale bar, 100 µm. White arrows indicate EdU-positive cells. **f**, NEFA quantification (mmol/plasma total protein quantification) in fed and fasted (48 h) 18-week-old fish, wild-type fed (*n* = 8), UBI:γ_1(R70Q)_ fed (*n* = 7), γ_1(R70Q)_ fed (*n* = 7), wild-type fasted (*n* = 8), UBI:γ_1(R70Q)_ fasted (*n* = 7), and γ_1(R70Q)_ fasted (*n* = 7). **g**, Blood triglyceride quantification (mg dl^−1^) in fed and 48 h fasted 18-week-old fish, wild-type fed (*n* = 6), UBI:γ_1(R70Q)_ fed (*n* = 7), γ_1(R70Q)_ fed (*n* = 7), wild-type fasted (*n* = 9), UBI:γ_1(R70Q)_ fasted (*n* = 8), and γ_1(R70Q)_ fasted (*n* = 7). **h**,**i**, Kaplan–Meier survival analysis of UBI:γ_1(R70Q)_ and γ_1(R70Q)_ lines, sex combined. Data are presented as mean ± s.d. (**e**–**g**). Significance was obtained by a one-way ANOVA followed by Tukey’s post hoc test (**e**), two-way ANOVA followed by Sidak test (**f**,**g**) and two-tailed log-rank calculation (**h**,**i**). Source data

**Extended Data Fig. 5:**
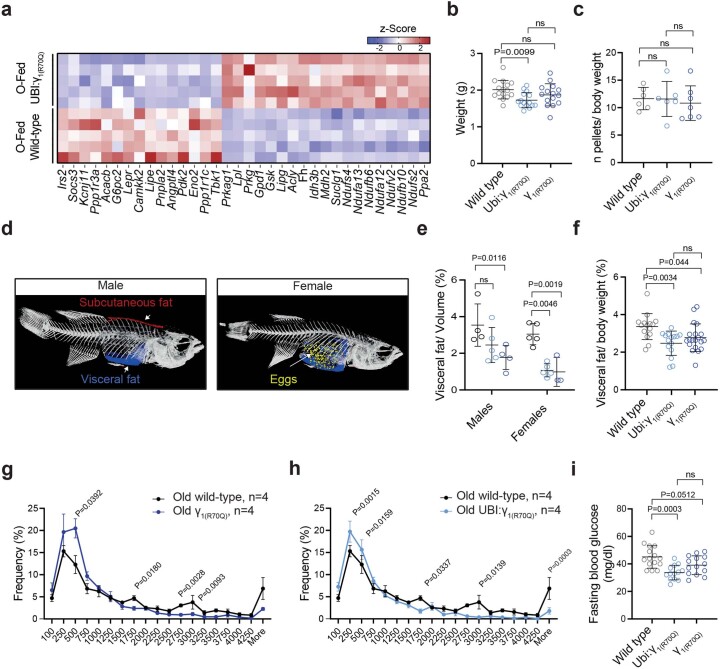
Sustained activation of AMPKγ1 promotes metabolic health and longevity. **a**) Z score heat map of genes involved in lipid and glucose handling. **b**) Body weight comparison in 18 weeks old wild-type (n = 15), UBI:_γ1(R70Q)_ (n = 15), and γ_1(R70Q)_ (n = 15) fish. **c**) Food consumption monitored over a week in 18 weeks old fish. Each dot represents the average value of pellets eaten/body weight by each fish. Old wild-type n = 6, UBI:_γ1(R70Q)_ n = 6, and γ1_(R70Q)_ n = 7. **d**) 3D Micro-CT analysis of whole-body composition in wild-type killifish. Visceral fat is indicated in blue, subcutaneous fat in red, and eggs in yellow. **e**) Micro-CT scan quantification of visceral fat in 18 weeks old fed wild-type and mutant lines (both sexes). Old wild-type males n = 4, UBI:γ_1(R70Q)_ males n = 5, and γ1_(R70Q)_ males n = 4, Old wild-type females n = 5, UBI:γ_1(R70Q)_ females n = 5, and γ1_(R70Q)_ females n = 3. **f**) Visceral fat quantification (mg of fat/body weight) of 18 weeks old wild-type (n = 12), UBI:γ_1(R70Q)_ (n = 15), and γ_1(R70Q)_ (n = 20) male fish. **g**, **h**) Frequency distribution of adipocyte area in 18-week-old wild-type, UBI:γ_1(R70Q)_, and γ_1(R70Q)_ fish, n = 4 fish/group. **i**) Fasting (48 h) blood glucose levels (mg/dL) of 18 weeks old negative transgenics (n = 15), UBI:γ_1(R70Q)_ (n = 12), and γ _1(R70Q)_ (n = 14) male fish. Data in **b**-**i** are presented as mean ± S.D. Significance was obtained by one-way ANOVA followed by Tukey’s post hoc test in **b**-**c**-**f**-**i**, Two-way ANOVA followed by Tukey’s post hoc test in E, and by two-tailed t-test in **g**-**h**. Source data

Our results above suggest that FLTP set wild-type adipose tissue metabolism in a quiescent state, whereas UBI:γ_1(R70Q)_ fish had no age-associated FLTP and exhibited elevated energy metabolism. Thus, we asked whether sustained energy metabolism could promote better tissue homeostasis late in life. Comparing the transcriptome profile of old fed UBI:γ_1(R70Q)_ and wild-type fish, we identified 2,963 DEGs (FDR < 0.05). KEGG pathway analysis revealed that the strongest upregulated categories were proteostasis, DNA replication and repair (Fig. 5a). Within these categories, we found upregulated genes involved in DNA synthesis, DNA double-strand break repair, including *Rad51*, *Xrcc1*-*2* and *Atm*, as well as many genes encoding proteasomal subunits (Fig. 5c), suggesting increased cellular proliferation, improved DNA damage surveillance and proteome turnover. Furthermore, upregulated genes were enriched for processes such as carbon metabolism and the TCA cycle (Fig. 5a), corroborating the sustained energy metabolism in UBI:γ_1(R70Q)_ fish late in life. We found upregulation of multiple cytosolic and mitochondrial ribosomal genes, possibly indicating enhanced protein translation. On the other hand, downregulated genes were enriched for insulin secretion, adipocytokine signaling and inflammatory processes (Fig. 5b). Here were found many genes whose expression positively correlates with obesity, insulin resistance, type-2 diabetes and chronic inflammation, such as *Irs2*, *Angptl4*, *Lepr*, *Camkk2* and *Tbk1* (Extended Data Fig. 5a). Moreover, a subset of downregulated genes is implicated in hypertrophic cardiomyopathy. Notably, γ2 activation has been shown to trigger genes implicated with cardiomyopathy^15^, whereas γ1 activation apparently antagonizes such genes, indicating a possible opposing role for these isoforms.

### AMPK_γ1_ activity promotes metabolic health and longevity

Next, we examined the health status of mutant lines. Old UBI:γ_1(R70Q)_ and γ_1(R70Q)_ fish showed a slight reduction in body weight compared to age-matched wild-type fish, though the latter did not reach statistical significance (Extended Data Fig. 5b). Notably, the food consumption of 18-week-old mutant fish monitored over 1 week was not different from old wild-type fish (Extended Data Fig. 5c). Consistent with human and mice data, we recently showed that killifish visceral adiposity expands as a function of age^31^, possibly due to the suppression of energy metabolism shown above. As such, we monitored the visceral adiposity in old wild-type and mutant lines. Body scan analysis (% of volume; Extended Data Fig. 5d,e) and visceral fat quantification (% of body weight; Extended Data Fig. 5f) revealed a general reduction of visceral adiposity in the R70Q-bearing lines compared to wild-type in both sexes, indicating a reduced age-related fat deposition.

**Extended Data Fig. 6:**
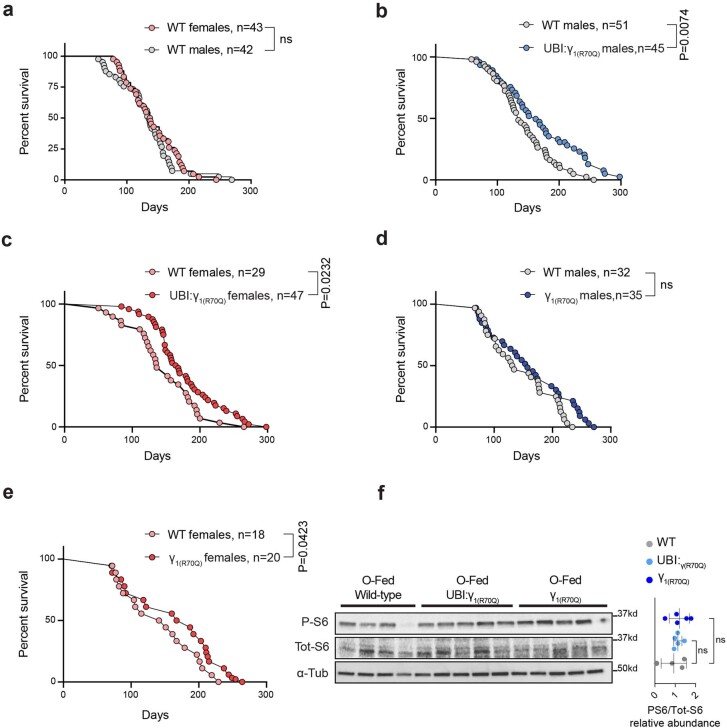
Sustained activation of AMPKγ1 promotes longevity. **a**) Survival analysis of GRZ-AD wild-type male and female single-housed fish. **b**) Survival analysis of UBI:γ_1(R70Q)_ compared to wild-type male fish. **c**) Survival analysis of UBI:γ_1(R70Q)_ compared to wild-type female fish. **d**) Survival analysis of γ_1(R70Q)_ compared to wild-type male fish. **e**) Survival analysis of γ_1(R70Q)_ compared to wild-type female fish. **f)** Representative Western blots and densitometric analysis of phospho and total ribosomal S6 in the adipose tissue of old fish. Old wild-type n = 4, UBI:γ_1(R70Q)_ n = 5, and γ1_(R70Q)_ n = 5. Statistical analysis was calculated by two-tailed log-rank in **a-e**, by one-way ANOVA followed by Tukey’s post hoc test in **f**. Source data

Adipogenesis, as defined by the new production of adipocytes, dramatically declines with aging, promoting lipodystrophy and metabolic dysfunction^32^. As transcriptomic analysis indicated a possible signature of increased cellular proliferation in UBI:γ_1(R70Q)_, we performed a pulse–chase ethynyl deoxyuridine (EdU)-labeling experiment to determine the proliferative status of the adipose tissue in old animals. Notably, both UBI:γ_1(R70Q)_ and γ_1(R70Q)_ lines showed a higher number of dividing cells compared to age-matched wild-type fish (Fig. 5d,e). In line with higher proliferative potential and expression of DNL genes, UBI:γ_1(R70Q)_ and γ_1(R70Q)_ fish displayed a higher number of small adipocytes and a reduced number of hypertrophic adipocytes relative to wild-type fish (Extended Data Fig. 5g,h). Overall, these data indicate that increased AMPK_γ1_ complex activity sustained adipose tissue turnover in old animals. It is notable that both UBI:γ_1(R70Q)_ and γ_1(R70Q)_ lines showed reduced serum triglycerides (TGs) and circulating NEFA levels under basal and fasting conditions, an effect that was stronger in UBI:γ_1(R70Q)_ (Fig. 5f,g). They also showed reduced fasting blood glucose levels (Extended Data Fig. 5i). Thus, chronic activation of the AMPK_γ1_ complex improves lipid and glucose parameters in old killifish. Finally, we determined the effect of sustained AMPK_γ1_ activation on lifespan. As no sex-related gap in life expectancy was observed in single-housed killifish in our husbandry conditions (Extended Data Fig. 6a), we used both sexes for survival analysis. The median survival of UBI:γ_1(R70Q)_ and γ_1(R70Q)_ (sexes combined) was 20% and 14% higher compared to wild-type fish, respectively (Fig. 5h,i). Analysis of each sex separately showed a 19.1% and 8% (the latter not statistically significant) increase in male and a 21.6% and 15.8% increase in female median lifespan of UBI:γ_1(R70Q)_ and γ_1(R70Q)_, respectively (Extended Data Fig. 6b–e), indicating a stronger effect in females.

AMPK is known to suppress the mTOR pathway and a modest reduction in mTOR activity promotes longevity across taxa^33–37^. We, therefore, wondered about the state of the mTOR pathway upon AMPK_γ1_ activation. To this end, we determined the phospho levels of the ribosomal S6 protein, a downstream mTORC1 target, in old wild-type and UBI:γ_1(R70Q)_ and γ_1(R70Q)_ fish; however, we saw no significant change in p-S6, potentially indicating an mTOR-independent longevity effect. This observation is in line with our RNA-seq analysis reflecting higher rather than lower protein translation and DNA synthesis. Collectively, our data reveal that the refeeding-associated AMPK_γ1_ complex stimulates tissue turnover, thus promoting health and longevity.

### *PRKAG1* expression is a marker of healthy aging in humans

**Fig. 6:**
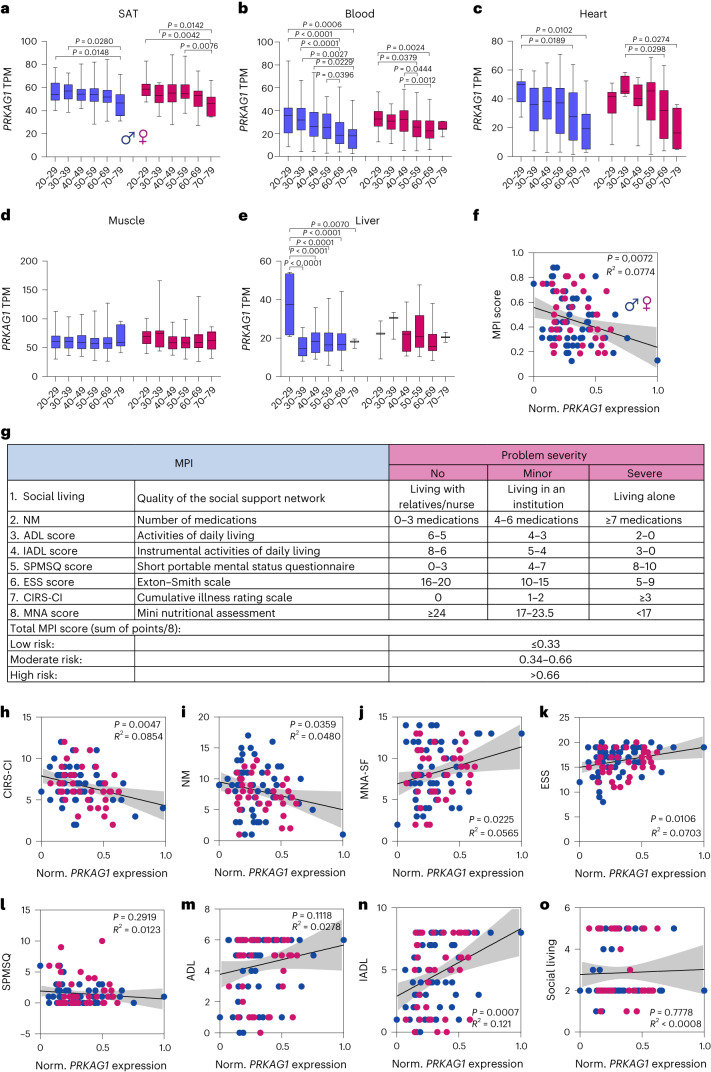
Human AMPK γ1 expression is a proxy of health in old individuals. **a**–**e**, Box-plots showing human normalized *PRKAG1*expression levels across age groups in decadal brackets. Box-plot minimum is the smallest value within the interquartile range (IQR) below the 25th percentile and maximum is the largest value within the IQR above the 75th percentile. Box-plot center is the 50th percentile (median) and box bounds are the 25th and 75th percentiles. Data are available in the GTEx Consortium (GTEx analysis v.8). For group numbers, see Supplementary Table 1. **f**, Pearson’s correlation between *PRKAG1*expression and MPI score in males (blue dots) and females (pink dots). The shadow represents the 95% confidence interval. **g**, Calculation of the MPI. **h**–**o**, Pearson’s correlation analysis between *PRKAG1*expression and MPI subdomains. The shadow represents the 95% confidence interval. Significance was obtained by one-way ANOVA followed by Tukey’s post hoc test (**a**–**e**) and two-tailed *P* values are reported (**f**,**h**–**o**). Source data

**Extended Data Fig. 7:**
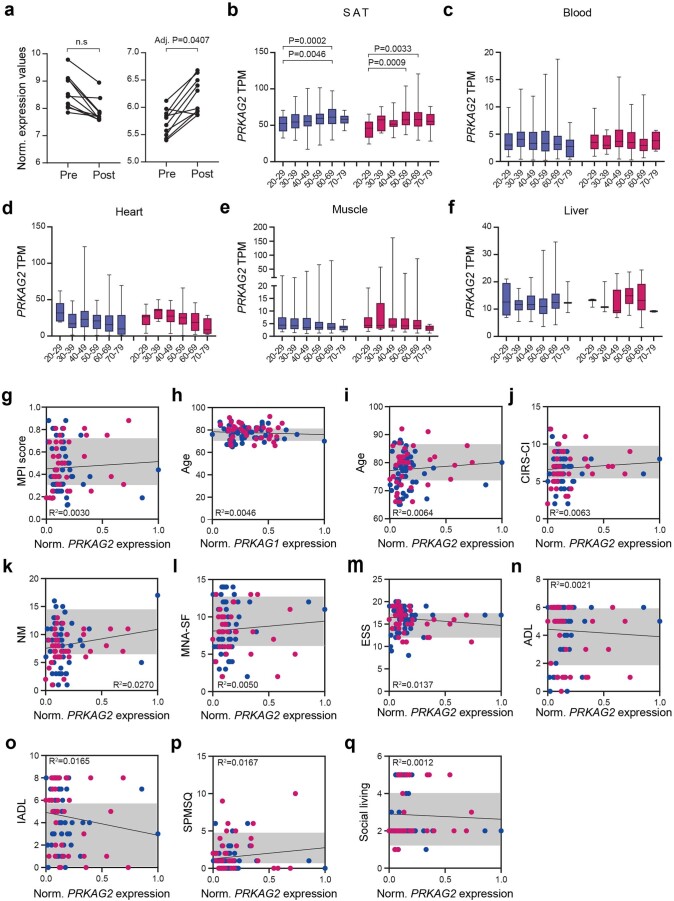
Human AMPK γ1 expression is a proxy of health in older individuals. **a**) GEO2R analysis using DESeq2 was performed to assess the expression of human *PRKAG1* and *PRKAG2* in subcutaneous adipose biopsies obtained both before (2 hours, pre-fasting) and after (26 hours, post-fasting) a fasting period, n = 10. Data from Defour et al.^23^. **b–f**) Box-plots showing human normalized *PRKAG2* expression levels across age groups in decadal brackets. Box-plot minimum is the smallest value within the interquartile range (IQR) below the 25th percentile and maximum is the largest value within the IQR above the 75th percentile. Box-plot center is the 50th percentile (median) and box bounds are the 25th and 75th percentiles. Data are available in the GTEx Consortium (GTEx analysis V8). For group numbers see Supplementary Table 1. **g**) Pearson’s correlation between *PRKAG2* expression and MPI score in males (blue dots) and females (pink dots). The shadow represents the 95% confidence interval. **h, i**) Pearson’s correlation between donor’s age and *Prkag1* (**h**) and *PRKAG2* (**i**) expression. **j–q**) Pearson’s correlation between *PRKAG2* expression and MPI-subdomains. The shadow represents the 95% confidence interval. Significance was obtained by GEO2R calculated adjusted P-value (Benjamini & Hochberg) in **a**, one-way ANOVA followed by Tukey’s post hoc test in **b**-**f**. Source data

Finally, we wished to evaluate the relevance of AMPK_γ1_ in human health and longevity. Defour et al.^23^ performed a transcriptome analysis of human subcutaneous adipose tissue (SAT) in response to fasting. In line with our killifish data, fasting generally upregulated *PRKAG2* and downregulated *PRKAG1* expression, though the latter did not reach statistical significance (Extended Data Fig. 7a), potentially indicating a nutritional-dependent expression of γ subunits in humans. We next used the human genotype tissue expression (GTEx) dataset to evaluate the expression of γ subunits as a function of age. Consistent with killifish data, *PRKAG1* expression significantly decreased as a function of age in SAT, blood cells, heart (in both sexes) and liver (in males only) (Fig. 6a–e). By comparison, *PRKAG2* expression significantly increased in SAT only (Extended Data Fig. 7b–f).

Blood showed one of the most substantial age-related *PRKAG1* downregulation among analyzed tissues. As such, we asked whether reduced expression of *PRKAG1* in the blood of older humans could be associated with multimorbidity, frailty and mortality risk. To this end, we determined the expression of *PRKAG1* and *PRKAG2* by qPCR in peripheral blood mononuclear cells (PBMCs) from 93 older donors (aged between 65 and 90 years). Study participants were patients hospitalized for non-neurological, non-surgical medical conditions. Notably, we established a cohort covering a broad range of age-related diseases with very heterogenous functional levels. For all participants, the multidimensional prognostic index (MPI) was recorded at the time of presentation to the hospital. The MPI is a validated prognostic model consisting of eight components, namely, Activities of Daily Living (ADL), Instrumental ADL (IADL), Short Portable Mental Status Questionnaire (SPMSQ), Cumulative Illness Rating Scale–Comorbidity Index (CIRS–CI), Mini Nutritional Assessment Short Form (MNA-SF), Exton Smith Scale (ESS), number of medications (NM) and the quality of the social support network (Fig. 6g). Thus, it is based on information regarding functional, psychosocial, clinical and nutritional status^38,39^. The MPI has been shown to predict older individuals’ mortality rate more accurately than the classical physical frailty score^39^. Higher values of MPI reflect poor health outcomes and higher mortality risk. We found that γ1, but not γ2, expression was inversely correlated with MPI score (Fig. 6f and Extended Data Fig. 7g); however, we observed no correlation between γ subunits expression and the age of the donors (Extended Data Fig. 7h,i), indicating that the relationship between MPI and γ1 expression is independent of the chronological age of the donors given this later stage life bracket. Looking at the individual MPI components, we observed a significant negative correlation of *PRKAG1* expression with CIRS–CI and NM (Fig. 6h,i). At the same time, MNA, ADL, IADL and ESS were positively correlated with *PRKAG1* expression (Fig. 6j–o), indicating reduced multimorbidity and improved nutritional status and functional skills in older individuals to be associated with higher γ1 expression. By contrast, *PRKAG2* expression was not associated with any MPI component (Extended Data Fig. 7j,q). These observations highlight the functional relevance of individual γ subunits and indicate that changes in *PRKAG1* expression are specific rather than a consequence of general changes in gene expression due to disease.

## Discussion

We determined the transcriptional response to food deprivation in young and old fish’s visceral adipose tissue (VAT). We found that aging promotes an FLTP that reduces metabolic activities, thereby setting VAT in a quasi-quiescent state; however, because we only measured FLTP in VAT, we do not yet know the full extent of FLTP in other tissues. Notably, we observed dysregulation of AMPK γ subunits in adipose and other aging tissues, implying that the presence of FLTP could be a broad response. In line with our data, Montesano et al. reported the orexigenic neuropeptide Y expression, normally induced by fasting, to progressively increase in killifish diencephalon (thalamic area) during aging^40^. These data indicate that FLTP might not be limited to the adipose and could reflect intrinsic tissue processes or a systemic effect of FLTP in adipose on other tissues.

Another critical question is whether FLTP is conserved throughout vertebrate species. Human aging is often accompanied by a reduction of subcutaneous adiposity, which progressively relocates into visceral depots^41^. Loss of SAT is associated with poor health outcomes in humans, yet the reasons remain unknown. In line with FLTP observed in killifish, age-related changes in human SAT include suppression of energy metabolism, adipogenesis^32^ and an increased number of infiltrating immune cells^42^. In addition, older individuals often exhibit elevated plasma NEFAs^43,44^ and increased liver steatosis^45^, both signs of an ongoing fasting response. Finally, we could show a broad age-associated reduction of γ1 expression in human SAT. Altogether these findings suggest the possible presence of FLTP in human SAT, which could reflect the suppression of self-renewal processes and promotion of elevated plasma lipids species. Another organ where FLTP might take place is skeletal muscle, as age-related sarcopenia is characterized by the inability of the muscle to maintain the correct level of protein synthesis in response to feeding or exercise, a condition defined as anabolic resistance^46^. Thus, multiple lines of evidence support the notion that aging is associated with a constitutive fasting-like response across vertebrate species.

Mechanistically we demonstrated that the AMPK_γ1_ complex is a key mediator of the age-related fasting-like response. γ1 is downregulated during fasting and aging but its chronic activation prevents FLTP and sustains energy metabolism in older age, thus, improving adipogenesis, metabolic health and promoting longer life. Our data provide some of the first concrete genetic evidence that AMPK in general and AMPK_γ1_ complex causally and specifically extends vertebrate lifespan, as most other studies infer a role based on metformin^47^, a drug with multiple targets. Furthermore, the evidence that γ1 anticorrelates with multidimensional frailty as measured by the MPI score, indicates AMPK_γ1_ complex as a hallmark of healthy aging and robustness in humans.

Sustained AMPK_γ1_ activation enhances the lifespan of both sexes in killifish. Notably, this effect occurs without suppressing mTOR activity while concurrently promoting tissue self-renewal. A possible explanation could be that AMPK_γ1_ maintains tightly connected energy metabolism to tissue turnover late in life, thereby promoting rejuvenation. This is in line with the evidence that increased energy metabolism promotes longevity. For instance, GH-deficient and SIRT6-overexpressing mice exhibit higher energy metabolism and longer life compared to wild-type mice^48,49^. Furthermore, increased OXPHOS late in life stimulates muscle regeneration by activating satellite cells’ awakening and proliferation^50^. On the other hand, longitudinal human studies indicate that longevity correlates with reduced energy metabolism. Conceivably, energy homeostasis must be at some optimum to conserve resources yet sustain anabolism to promote organismal longevity.

Our data indicate that fasting induces γ2 and suppresses γ1, whereas refeeding induces γ1 and suppresses γ2 in multiple tissues, suggesting that the different γ subunits mediate different metabolic states. Consistently, activation of AMPK_γ2_, but not AMPK_γ1_^51^, results in metabolic disorders^14^. Furthermore, γ2 seems to have a nuclear localization, whereas γ1 is cytosolic^52^. Conceivably, the binding to one or the other γ subunit confers to the AMPK complex different cellular localization or substrates interaction.

Of note, γ1 seems to be induced by feeding, which apparently contradicts the notion that AMPK reacts in response to energy deprivation. In fact, the AMPK complex is inhibited by high ATP levels and activated by increased AMP/ATP ratio or low-glucose concentrations^53,54^; however, the evidence that γ1 expression is low under fasting and high under food availability indicates that this subunit allows AMPK to keep functioning under high-energy conditions. Notably, among all the γ subunits, γ1 only is sensitive to ADP, whose levels are considerably higher compared to AMP under normal feeding conditions^55^. While prolonged fasting suppresses energy metabolism, refeeding boosts it. Nevertheless, during this transition, several AMPK-dependent processes, such as turnover of cellular components, mitochondrial biogenesis and revival of energy metabolism are required. Conceivably, the switch from γ2 to γ1 could be an important step in this transition.

By contrast, the γ2 subunit seems to be insensitive to ADP stimulation but is the most responsive to AMP fluctuations under low ATP concentrations^55^. Consistently, we show that the AMPK γ2 subunit is induced under fasting conditions in young healthy fish, whereas studies in mice and humans showed AMPK γ2, but not γ1, activation stimulates hyperphagia^14^ and bradycardia^56^, conditions associated with fasting. This could indicate that AMPK_γ2_ function is physiologically required exclusively upon fasting and explains why, unlike AMPK_γ1_, chronic activation of AMPK_γ2_ leads to metabolic dysfunction in normally fed mice or humans. Astre et al. showed that mutation in the *APRT* gene, a key enzyme in AMP biosynthesis, mimics a caloric-restriction effect, inducing lifespan extension in killifish males^57^. This effect was associated with higher expression of the γ2 subunit. This strengthens the evidence of AMPK_γ2_ to be physiologically required only under energetic stress conditions and implies that various DIs might represent a concrete strategy to counter the pathological effect of AMPK_γ2_ hyperactivity in humans.

The evidence that different AMPK complexes mediate different metabolic states highlights the potential importance of complex composition as a new layer of regulation in defining AMPK functions. This is particularly important for drug-screening designs. In fact, AMPK-activating compounds are identified based solely on their ability to stimulate the kinase activity, but very often, without considering the nature of the complex activated. The lack of selectivity could result in the simultaneous activation of different AMPK complexes with mixed effects on the body’s physiology and metabolism. Yet, compounds that selectively target AMPK_γ1_ are unknown, thus, further studies are required to identify and test their effect on aging, robustness and longevity of vertebrate species.

We wanted to understand the potential role of the γ1 subunit in the refeeding phase. Refeeding reverses the effects of fasting, increases energy expenditure and stimulates anabolic metabolism, thereby promoting tissue growth and regeneration. Conceivably, the γ1 subunit is important to restart energy metabolism after prolonged fasting to sustain the re-activation of high-energy demanding processes such as DNA synthesis and protein translation. This is in line with mice studies, where AMPK_γ1_ gain-of-function mutants exhibit higher whole-body energy expenditure on a chow diet^51^. On the other hand, reduced γ1 would lead to an inadequate refeeding response due to unmatched energy demand and supply. Though we have not explicitly investigated the role of γ1 under refeeding, we observed that old fish with reduced γ1 expression showed a transcriptome profile similar to that of fasted fish. Consistently, adipose tissue-specific AMPK knockout mice exhibit reduced energy expenditure and oxygen consumption under normal feeding conditions^58^, indicating that AMPK regulates energy metabolism also under feeding conditions. Further studies will be necessary to fully explore the role of AMPK_γ1_ during refeeding.

The evidence that refeeding-associated factors such as AMPK_γ1_ become impaired in older individuals could have important implications for anti-aging interventions such as dietary restriction (DR) or intermittent fasting (IF). In fact, the protective effects mediated by either DR or IF derive from the coupled effects of fasting and refeeding that constrain the metabolism to shift from catabolism to anabolism periodically. The evidence that older animals might become refractory to the refeeding arm implies that DIs initiated late in life might exacerbate catabolic activities or fail to reinstate the proper anabolic profile upon refeeding and ultimately promote tissue wasting. In line with this idea, we showed that older individuals, as opposed to young, displayed an enhanced inflammatory signature upon food deprivation. Thus, selective stimulation of AMPK_γ1_ could represent a potential strategy to reinstate the beneficial response of a late-life DR through the maintenance of a correct feeding response.

In conclusion, our study revealed that age-associated metabolic quiescence can be prevented by selective stimulation of an AMPK_γ1_ complex that in turn promotes metabolic health and longevity in vertebrate species.

## Methods

### Fish husbandry

All experiments were performed on adult (young, aged 6–8 weeks; and old, aged 18–20 weeks) African turquoise killifish *N.* *furzeri* laboratory strain GRZ-AD. Adult fish were raised singularly in 2.8-l tanks from the second week of life. Water parameters were pH 7–8, kH 3–5 and temperature of 27.5 °C. The system automatically replaced 10% of the water with fresh water daily. Fish were raised in 12 h of light and 12 h of darkness and fed with 10 mg of dry pellet (BioMar INICIO Plus G) and Premium Artemia Coppens twice a day (for a total amount of food daily delivered equal to 2–3% of fish weight). The first feeding was made at 8:30 and the second at 13:30. For tissue collection, fish were killed by rapid chilling. Tissues were rapidly extracted by dissection, snap-frozen in liquid nitrogen and stored at −80 °C. Five days of fasting were experimentally validated by monitoring blood glucose levels, which started declining between 24 and 48 h and leveled off at 5 d of fasting under our husbandry conditions. This potentially corresponds to the near depletion of liver glycogen storage. Animal experiments were carried out in accordance with relevant guidelines and approved by ‘Landesamt für Natur, Umwelt und Verbraucherschutz Nordrhein-Westfalen’, 81-02.04.2019.A055.

### Generation of the UBI:γ_1(R70Q)_ line

The Tol-2 UBI:H2ACFP-(2x)SV40pA/cmlc2:eGFP vector, originally generated using the TOL-2 kit^59^, was digested with KpnI to excise H2ACFP. The *Prkag1(R70Q)* sequence was generated by PCR-mediated site-directed mutagenesis using the killifish *Prkag1* cDNA as a template. *Prkag1(R70Q)* was then recombined with UBI:H2ACFP-(2x)SV40pA, previously digested with KpnI to excise H2ACFP, using the NEBuilder HiFi DNA Assembly Cloning kit (primers are listed in Supplementary Table 2). The resulting plasmid (UBI:_γ1(R70Q)_) was then amplified in *Escherichia* *coli* and purified with Wizard Plus SV Midipreps DNA Purification Promega. Tol-2 transposase messenger RNA was synthesized by in vitro transcription using the mMESSAGE mMACHINE SP6 (Ambion) kit and the pCS2FA plasmid, previously linearized with NotI, as a template. The mRNA was then purified by lithium chloride precipitation, aliquoted and stored at −80 °C. Transgenic fish were generated by injecting 1–2 nl of a solution containing 30 ng μl^−1^ Tol-2 mRNA, 40 ng μl^−1^ UBI:_γ1(R70Q)_ plasmid and 0.4 M KCl into one-cell stage *N.* *furzeri* embryos and 1% phenol red was used as a visual control for successful injections. Potential founders expressing a myocardium-specific eGFP signal were identified under a fluorescent microscope and backcrossed with the GRZ-AD wild-type strain for three generations. To map the transposon insertions, we used a PCR-based sequencing approach, where forward primers hybridizing at the edges of the Tol-2 arms were coupled with degenerate reverse primers to amplify DNA spanning the transposon insertion junctions (Extended Data Fig. 7a; Supplementary Table 2 contains primer sequences). All the resulting bands were purified, sequenced and mapped onto the killifish genome using BLAST. Each integration site was cross-validated using another couple of primers encompassing the transposon insertion junctions (Extended Data Fig. 7a). We obtained a stable transgenic line carrying two copies of UBI:_γ1(R70Q)_: one integrated into the third intron of *mybl* and the other into the first intron of an unidentified gene *LOC107386866* (Extended Data Fig. 7b). We used this dual copy line as a further reduction of copy number resulted in weak transgenic expression in several tissues (data not shown). Fish having two copies of the transgene in heterozygous and homozygous configurations induced overexpression of *Prkag1* of about five- and tenfold in the fin (Extended Data Fig. 7c). The mRNA expression of resident genes at the integration site seemed unaffected by the transgene insertion (Extended Data Fig. 7d). Negative transgenic fish (defined as wild-type in the text) were used as control fish in aging comparative and survival analysis.

### γ_1(R70Q)_ and γ1(−/−) line generation

CRISPR/Cas9 genome editing was performed according to previous work^60^. All the single guide RNAs (sgRNAs) were designed based on the CHOP-CHOP web-based tool (https://chopchop.cbu.uib.no). The same sgRNA was used to generate both γ_1(R70Q)_ and γ1(−/−) mutant lines. The single-strand DNA (ssDNA) to generate γ_1(R70Q)_ was designed to contain 45 bp of homology arm and two base-pair mutations in the region encompassing the coding sequence for γ1 amino acid residue R70. Alt-R S.p. HiFi Cas9 Nuclease, Alt-R sgRNA and the ssDNA template were purchased from IDT (Supplementary Table 2 provides sequences). One-cell-stage embryos were injected with 1–2 nl of a solution containing Cas9 enzyme (200 ng µl^−1^), sgRNA (20 ng µl^−1^), ssDNA (40 ng µl^−1^), KCl (0.2 M) and 1% phenol red. The F0 generation was genotyped by fin-clipping to identify potential founders (Extended Data Fig. 7e,f; Supplementary Table 2 contains primers sequences). Selected founders were then backcrossed with the GRZ-AD strain for four generations to reduce the potential presence of background mutations induced by CRISPR editing.

### Food consumption and Fulton’s index analysis

To monitor food consumption, single-housed fish were fed daily with a fixed amount of food (*n* = 40 dry pellets per fish) using a handheld dispensing machine (SDH-1, XQ instruments). Fish were then allowed to eat for 1 h. During this time, the water flow was interrupted to prevent food from washing away. At the end of this time, the bottom of every tank was recorded for about 15 s by a photo camera (SQ12 spy mini DV camera, RC-group) positioned above the lid. Videos were analyzed to count the number of leftover pellets (LPs). Food consumption was calculated as (40 − LP)/g of body weight. Fulton’s index (*K*) was calculated as (weight × 100)/length^3^.

### MicroCT body scan analysis

Killed fish were scanned using a high-performance in vivo microCT scanner SkyScan 1176 (Bruker) with an isotropic voxel size of 18 µm^3^ using the following parameters: voltage 45 kV and current 475 µA, using a 0.5-mm aluminum filter and exposure time of 260 ms. All scans were performed over 360 degrees with a rotation step of 0.6 degrees and a frame averaging of 2. Image data were reconstructed using the NRrecon Software (Bruker) with the following parameters: smoothing degree 4, ring artifact reduction 3, beam hardening correction 30% and defect pixel masking 5%. Gray values of all images were standardized by setting the contrast range of the histogram from 0 to 0.03. Relative subcutaneous, visceral fat and eggs yolk quantification from reconstructed images were determined using the CTAn software (Bruker).

### Tissue fixation, H&E staining, immunofluorescence, EdU labeling, adipocyte area counting and mtDNA content

Killifish were killed by rapid chilling and immersed in formalin solution for 72 h at 4 °C after the visceral cavity was opened and the gill opercula were removed. Fish were transferred to EDTA solution (500 mM, Ambion) for another 72 h and paraffin embedded. Next, 4-µm paraffin sections were made and stored at room temperature. The slides were deparaffinized and stained with H&E using the Gemini station (Thermo Scientific, A81510100). Heat-mediated antigen retrieval was initially used on deparaffinized slides for immunolabelling to break protein crosslinks. Next, slides were washed thrice in 1 × PBS + Triton 0.3%, blocked for 1 h at room temperature with a solution of goat normal serum 10% (Abcam, ab748) and BSA 2.5% and incubated with rabbit anti-L-plastin primary antibody (1:400 dilution, GTX124420, Genetex) overnight at 4 °C. The next day, slides were washed three times in PBST and incubated first with the SignalStain Boost IHC Detection Reagent (8114S, CST) for 30 min at room temperature, then with the DAB substrate for 1–2 min. Finally, slides were counterstained with hematoxylin, dehydrated with alcohol and xylene, and mounted using Fluoromount-G with 4,6-diamidino-2-phenylindole (DAPI) (00-4959-52, Thermo). Images were acquired using a Nikon eclipse Ci microscope. To label dividing cells, fish were intraperitoneally injected with 8 µg g^−1^ of the weight of EdU 6 h before killing. EdU detection on deparaffinized slides was performed using the Click-iT EdU Alexa Fluor 488 Imaging kit according to the manufacturer’s instructions (C10337, Thermo). Images were acquired using a Leica DMI6000B microscope (software Leica application suite x3.5.7.23225). The frequency distribution of adipocyte areas was obtained using Adiposoft software (v.1.16)^61^. MtDNA copy number was determined by qPCR with primers amplifying the mitochondria 16S rRNA and the nuclear gene locus *Cdkan2a/b* according to Hartmann et al.^62^.

### Adipose tissue dissociation protocol

Adipose tissues were dissociated by adapting a protocol from Bresciani et al.^63^ with small modifications. In brief, freshly extracted adipose tissues were washed twice in PBS (1×), incubated with 500 µl dissociation mix (0.25% trypsin–EDTA (Gibco, 25200-056), collagenase (4 mg ml^−1^, Sigma, C9891) and DNase (100 ng ml^−1^, Sigma, DN25-10MG)). Samples were placed in a heat block at 30 °C for about 45 min and harshly pipetted until fully homogenized. Afterwards, 800 µl DMEM–10% FBS were added to the dissociation mix and samples were centrifuged 5 min at 400 r.c.f. Pellets were then washed twice with 800 µl DMEM–10% FBS, finally resuspended in 500 µl XF Base Medium Minimal DMEM (Agilent Technologies), filtered through a 70-µm nylon mesh into 50-ml tubes and used for downstream applications.

### Bioenergetics measurements

The analysis was performed using the Seahorse XFe96 (Agilent Technologies) adapting a protocol from van der Windt et al.^64^. In brief, freshly extracted single cells from the adipose tissue were plated in technical triplicates in a poly-d-lysine-coated XF cell culture microplate in XF Base Medium Minimal DMEM (Agilent Technologies) supplemented with glucose 10 mM, pyruvate 1 mM and l-glutamine 2 mM (180 μl final well volume). Cells were acclimated for 60 min at 28 °C after spinning down for 5 min at 400 r.c.f. before measurements. The Seahorse XF Cell Mito Stress Test kit (Agilent Technologies) was used to assess mitochondrial function. OCR and ECAR were measured at 28 °C under basal conditions and after drug injections. The drug injection order and final well concentration were (1) oligomycin 2 μM, (2) FCCP 0.5 μM and (3) rotenone/antimycin A 0.5 μM. Each measurement was performed three times, with a mix for 3 min, wait for 2 min and measure for 3 min. OCR and ECAR values were normalized to total protein concentration.

### Total RNA extraction and qPCR analysis

Killifish tissues were thawed in RLT buffer (QIAGEN) with 1% β-ME *v*/*v* and mechanically crushed in a mortar and pestle. They were crushed by plastic beads using Tissue Lyser LT (QIAGEN) at 50 oscillations per second for 15 min at 4 °C. Samples were then allowed to settle for 15 min on ice before centrifugation at 16,000 r.c.f. for 10 min at 4 °C. The supernatant was collected for subsequent RNA extraction using the RNeasy Mini kit (QIAGEN) according to the manufacturer’s instructions. The optional DNase step was always performed using the RNase-Free DNase Set kit (QIAGEN) according to the manufacturer’s instructions. The concentration and purity of the RNA were measured by NanoDrop. cDNA was generated using iScript (Bio-Rad). qPCR with reverse transcription was performed with Power SYBR Green (Applied Biosystems) on a ViiA 7 Real-Time PCR System (Applied Biosystems). Four technical replicates were averaged for each sample per primer reaction. EIF3C was used as an internal control for killifish samples, B-actin for human samples (Supplementary Table 2 details primers sequences).

### Transcriptomic profiling and computational analysis

VAT from fully fed or fasted male fish was collected for expression profiling. To reduce possible batch effects or variability due to circadian rhythms, fish were killed all at once within 2 h in the early afternoon. Collected tissues were snap-frozen in liquid nitrogen and stored at −80 °C. RNA extraction of all samples was performed at the same time. About 1 μg of total RNA was used per sample for library preparation. The ribosome removal step was conducted using the RiboZero rRNA removal kit (Illumina). The sequencing was performed on Illumina HiSeq4000 sequencing system (∼30 million reads per sample) using a paired-end two × 100-nt sequencing protocol. After removal of rRNA and tRNAs, reads were pseudo aligned to the reference genome (Nfu_20140520) using Kallisto (v.0.45.0)^65^. Genes with fewer than ten overall reads were removed. After normalization of read counts by making use of the standard median ratio for estimation of size factors, pairwise differential gene expression was performed using DESeq2 (v.1.24.0)^66^. The log_2_ fold changes were shrunk using approximate posterior estimation for GLM coefficients. The KEGG pathway analysis of significant genes was performed using ShinyGO v.0.76.2 (ref. ^67^). An FDR value < 0.05 was considered to be significant. Hierarchical clustering was calculated using FLASKI (https://flaski.age.mpg.de/).

### Blood glucose, triglyceride and free fatty acid quantification

Killifish were killed by rapid chilling and then transferred on a piece of gauze to carefully dry off their body. A small incision on the lateral side nearby the caudal fin was made using a steel blade. The incision was deep enough to penetrate skin and muscle and reach the aorta to release the blood. Then, 2 µl blood was immediately used to determine blood glucose concentration (mg dl^−1^) using the glucometer Accu-check guide (Roche). In contrast, 8 µl was used to determine blood TG concentration (mg dl^−1^) using Accutrend-plus (Roche). The excess blood was collected in a 1.5-ml tube. To avoid hemolysis and coagulation, blood was immediately mixed with 0.5 *v*/*v* heparin (3 mg ml^−1^ in 1 × PBS) and kept on ice for 5–10 min. Samples were then centrifuged for 10 min at 8,000 r.c.f. Afterwards, the plasma was collected, transferred to a new 1.5-ml tube and used directly for analysis or flash-frozen in liquid nitrogen and stored at –80 °C. Then, 8 µl plasma was used to determine NEFA concentrations using the Free Fatty Acid Quantitation Kit (Sigma, MAK044) according to the manufacturer’s instructions. NEFA values were expressed as nmol per plasma total protein concentration.

### Western blot analysis

RIPA buffer supplemented with cOmplete Protease Inhibitor (Roche) and PhosSTOP (Roche) was used for total protein extraction. Samples were lysed in RIPA buffer using Bioruptor Sonication System (Diagenode) for 20 min, then kept on ice for another 15 min. Afterwards, samples were centrifuged for 10 min at maximum speed to remove cell debris and the supernatants were transferred to new tubes. Protein concentration was estimated using micro-BCA Protein Assay kit (Thermo Fisher Scientific). Protein samples were then heated to 75 °C for 15 min in 2× Laemmli buffer with 0.9% 2-mercaptoethanol. Next, 20–25 µg of protein per sample was loaded on 4–15% MiniPROTEAN TGXTM Precast Protein Gels (Bio-Rad), and electrophoresis was performed at constant voltage of 200 V for about 30–40 min. After gel separation, proteins were transferred on nitrocellulose membranes using Trans-Blot TurboTM Transfer System (Bio-Rad) and blocked for 1 h with 5% BSA in 1× TBST. Afterwards, the membranes were incubated overnight with primary antibodies and for 1 h with the secondary antibody. Imaging and signal quantification of the membranes was performed with ChemiDoc Imager (Bio-Rad, software, Image Lab v.6.1). Western Lightning Plus Enhanced Chemiluminescence Substrate (PerkinElmer) was used as the chemiluminescence reagent. The following details the antibodies and relative dilutions used in this study. Total AMPKα (CST, 2532; 1:1,000 dilution), phospho(Thr172)-AMPKα (CST, 2535; 1:1,000 dilution), total AMPKβ (CST, 4150; 1:1,000 dilution), total-ACC (CST, 3676; 1:500 dilution), phospho(Ser79)-ACC (CST, 3661; 1:1,000 dilution), γ2 (Invitrogen, PA522331; 1:1,000 dilution), OXPHOS cocktail (Abcam, ab110413; 1;1,000 dilution), anti-α-tubulin (Sigma, T9026; 1:10,000 dilution), total ribosomal S6 (CST, 2317, 1:1,000 dilution), phospho(ser235) S6 (CST, 2211, 1:1,000 dilution), anti-mouse HRP (Thermo Fisher, G-21040; 1:5,000 dilution) and anti-rabbit HRP (Thermo Fisher, G-21234; 1:5,000 dilution).

### Lifespan analysis

All eggs used for survival analysis were collected daily from aged-matched parents within 10 d. After hatching, larvae were housed together (four larvae per 1.1-l tank) until they reached 3 weeks of age, then they were single-housed in 2.8-l tanks for the remaining lifespan. Fish mortality was scored starting in the sixth week when sexual maturation was fully reached. At this point, fish exhibiting reduced size, incomplete sexual maturation or body malformations were removed from the cohorts. Both males and females were used for the experiments. Fish were examined daily for signs of ill health. Senescent fish (>28 weeks) that showed signs of distress such as severe lethargy, anorexia or advanced sarcopenia were killed for humane reasons. The age at which an ill fish was killed was taken as the last available estimate of its natural lifespan. Survival curves were calculated using the Kaplan–Meier estimator. Statistical significance was calculated by the log-rank test.

### Patient population and isolation of human PBMCs

The study recruited participants ≥65 years of age who presented to the emergency department at the University Hospital Cologne. Approval was obtained from the institutional review board of the University of Cologne (EK20-1346 and EK19-1275) and written informed consent was obtained from all patients. The study was conducted in accordance with the Declaration of Helsinki and the good clinical practice guidelines by the International Conference on Harmonization and was registered in the German Clinical Studies register (DRKS00024592 and DRKS00017365).

In brief, heparinized whole blood was diluted 1:1 with PBS in sterile conditions and transferred to a Leucosep tube (Greiner Bio-One, cat no. GREI163290_500). Following centrifugation, a layer of PBMCs became visible and was carefully aspirated. PBMCs were then counted and assessed for viability using Trypan blue staining. Subsequently, PBMCs were cryopreserved in liquid nitrogen.

### RNA extraction from human PBMCS

Aliquots of 1 × 10^6^ viable PBMCs were thawed on ice. RNA was extracted employing a bead-based approach on a KingFisher Flex Magnetic Particle Processor (Thermo Fisher) using the MagMAX mirVana Total RNA Isolation kit (Thermo Fisher, A27828) according to the manufacturer’s instructions. The concentration and purity of the RNA were measured by NanoDrop.

### MPI

The MPI was calculated based on a comprehensive geriatric assessment. The MPI included clinical, cognitive, functional, nutritional and social parameters and was carried out using six standardized scales (ESS, IADL, ADL, CIRS, MNA-SF and SPMSQ), as well as information on the NM and social support network, for a total of 63 items in eight domains. An MPI was calculated from CGA as described previously, expressing it as a score from 0 to 1, being subdivided into three MPI groups, where MPI-1 (robust), 0–0.33; MPI-2 (pre-frail), 0.34–0.66; and MPI-3 (frail), 0.67–1.0.

### Statistics and reproducibility

The specific sample sizes can be found in the figure legends. We did not use a statistical method to predetermine the sample size; however, they are consistent with those reported in previous studies on the same topic^47,51,56,57,62^. Fish were indiscriminately allocated to groups for all experiments and no data were excluded from our study. For pairwise analyses, data distribution was never assumed to be normally distributed and statistical tests were conducted using nonparametric methods (Mann–Whitney *U*-test). In the case of multiple comparisons, we used a one-way ANOVA with Tukey’s post hoc correction for normally distributed data and a Kruskal–Wallis test with a Dunn’s post hoc test for data presumed to deviate from normality. A significance threshold of *P* < 0.05 was applied consistently throughout the study. All histological and survival analyses were conducted in a blinded manner.

### Reporting summary

Further information on research design is available in the Nature Portfolio Reporting Summary linked to this article.

### Supplementary information

Supplementary Information Supplementary Information Table 1, Supplementary Information Table 2.

Reporting Summary

### Source data

Source Data Fig. 1 Statistical source data.

Source Data Fig. 2 Statistical source data.

Source Data Fig. 3 Statistical source data.

Source Data Fig. 3 Unprocessed western blots.

Source Data Fig. 4 Statistical source data.

Source Data Fig. 5 Statistical source data.

Source Data Fig. 6 Statistical source data.

Source Data Extended Data Fig. 1 Statistical source data.

Source Data Extended Data Fig. 2 Statistical source data.

Source Data Extended Data Fig. 2 Unprocessed western blots.

Source Data Extended Data Fig. 3 Statistical source data.

Source Data Extended Data Fig. 3 Unprocessed western blots.

Source Data Extended Data Fig. 4 Statistical source data.

Source Data Extended Data Fig. 4 Unprocessed western blots.

Source Data Extended Data Fig./Table 5 Statistical source data.

Source Data Extended Data Fig. 6 Statistical source data.

Source Data Extended Data Fig. 6 Unprocessed western blots.

Source Data Extended Data Fig. 7 Statistical source data.

**Extended Data Fig. 8:**
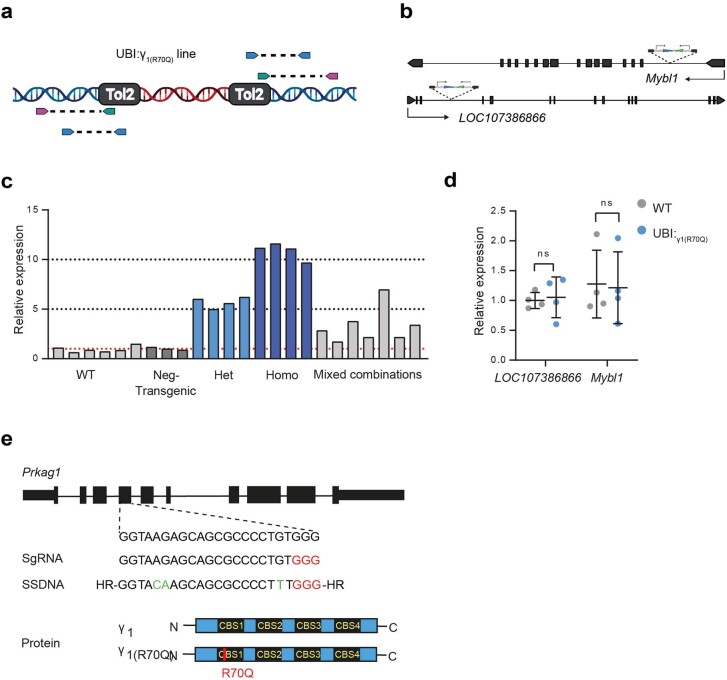
Generation of Prkag1 mutant lines. **a**) Schematic representation of the transposon integration mapping strategy. The red DNA fragments represent the UBI:γ_1(R70Q)_ expression cassette, pink arrows represent degenerate primers that anneal to unknown flanking genomic regions. **b**) Representation of the Tol-2 expression cassette integration loci. **c**) Fin clip qPCR analysis of *Prkag1* mRNA expression from fish with different copies of UBI:γ_1(R70Q)_ expression cassette. Each bar represents an independent biological replicate. **d**) qPCR analysis of *mybl1* and *LOC107386866* mRNA expression in UBI:γ_1(R70Q)_ relative to wild-type fish. n = 4 fish/group. **e**) Generation of γ_1(R70Q)_ CRISPR mutant line. Location of the sgRNA successfully targeting *Prkag1* exon 4 (Top). Green highlights the precise editing of specific codons introduced by the ssDNA template leading to the R70Q mutation (bottom). Protospace adjacent motif (PAM) is indicated in red. Data in **d** are presented as mean ± SD. Significance was obtained by two-tailed non-parametric Mann–Whitney U test. Source data

Source Data Extended Data Fig. 8 Statistical source data.

## Data Availability

All raw RNA-sequencing data can be found in the SRA database under BioProject ID PRJNA817434. Source data are provided with this paper. The reference killifish genome used in this study is Nfu_20140520. All raw RNA-sequencing data for human samples can be found in the GTEx database (https://gtexportal.org/home/datasets). All other data underlying this study will be provided by the corresponding author upon reasonable request.
