# Supplementary material for: Refeeding-associated AMPKγ1 complex activity is a hallmark of health and longevity
Source: Nat Aging. 2023 Nov 13;3(12):1544–60. doi: 10.1038/s43587-023-00521-y (PMC10724066; doi:10.1038/s43587-023-00521-y)
Supplement: Supplementary file 1 — Supplementary Information Table 1, Supplementary Information Table 2. [file 43587_2023_521_MOESM1_ESM.pdf]

nature aging

Article https://doi.org/10.1038/s43587-023-00521-y

# Refeeding-associated AMPK$_{\gamma 1}$ complex activity is a hallmark of health and longevity

In the format provided by the authors and unedited

**SI data table 1:** Sample sizes relative to Figure 6a-e and ext. data figure 7b-f

| Organ | Gender | 20-29y | 30-39y | 40-49y | 50-59y | 60-69y | 70-79y |
|---|---|---|---|---|---|---|---|
| SAT | Male | 34 | 42 | 61 | 143 | 147 | 13 |
| | Female | 18 | 15 | 41 | 68 | 67 | 8 |
| BLOOD | Male | 45 | 47 | 68 | 158 | 167 | 16 |
| | Females | 23 | 21 | 44 | 76 | 82 | 7 |
| MUSCLE | Male | 45 | 48 | 73 | 172 | 185 | 20 |
| | Females | 22 | 17 | 51 | 83 | 79 | 8 |
| LIVER | Male | 4 | 13 | 24 | 60 | 56 | 3 |
| | Females | 3 | 3 | 11 | 23 | 23 | 2 |
| HEART | Male | 12 | 17 | 44 | 107 | 104 | 10 |
| | Females | 10 | 9 | 22 | 47 | 46 | 4 |

**SI data table 2:** List of primers, sgRNA, and ssDNA.

| Killifish Real-time qPCR primers list | | |
|---|---|---|
| Name | **Forward** | **Reverse** |
| *Eif3c* | ggttaccagcagaagcagtc | tgttgaggaatgaagacgacg |
| *Fasn* | cgacgcacacgatgtctacaa | gtccactccagcttcccact |
| *Srebf1* | gcatctccaccagaacttcag | gccagctatgagacgattgttt |
| *Acaca* | tccagtacccatcctcgacacc | gcccagatcaaagaagccactctgcc |
| *Scd1* | cgctgacccccacaacgccaggc | ctgagctctagcttgcgcccc |
| *Acly* | ggagagattgggggcacagagg | cgaggtgaacatggtggcac |
| *Hspa5* | ctctgacaaaagacaatcacctgc | caaaggtcacttcaatttgagga |
| *Igf1r* | gacgaactgccaccatacgc | cagacgctgcccactgacac |
| *Pck1* | gagttctggcagaaagaggtg | cgtggtgggcaggtcactg |
| *Pdk2* | tcctaaaccaagtaccctccc | gcgactccatccaagcatcct |
| *Ppara* | agcagaggaaggctgaagc | catattttaggagagtcacctg |
| *Prkag1* | tccaggtgaagaaggcgttc | aaggagacttgtagtagcgatg |
| *Prkag2* | gtttgatgtgattaacctggctg | ctattctatccacgatagtttcc |
| **Humans Real-time qPCR primers list** | | |
| *b-Actin* | ctcttccagccttccttcct | agcactgtgttggcgtacag |
| *Prkag1* | gtatcactgtctgacatcctgc | ccctcaagtttcatctgattccc |
| *Prkag2* | caagccctgatcctcacaccagc | tacattcttaaccacttgcagcc |
| **Ubi:$\gamma_{1(R70Q)}$ cloning primers list** | | |
| 5' part *Prkag1* mRNA | ctatagggcgaattgggtacggtaccatgtaccc atacgatgttccagattacgctggaggtggaga gtgtattccagtaactattg | acaggggcgctgcttgtaccccgttagaaacaag |
| 3' part *Prkag1* mRNA | ttctaacggggtagcagcgcccctgtgggac | gccgctagatcatcatcgatggtaccttaggcggcg ccctcttc |
| **Tol-2 mapping primers** | | |
| Tol2 Left harm F | gattgcttttcagccccaaa | |
| Tol2 Right-harm F | aggtgctgtgcattgtggta | |
| Mapping random 1 R | gtacgagaatcgctgtcctNNNNNNNttagg | |
| Mapping random 2 R | gtacgagaatcgctgtcctNNNNNNNgtaca | |
| Mapping random 3 R | gtacgagaatcgctgtcctNNNNNNNaggta | |
| Mapping random 4 R | gtacgagaatcgctgtcctNNNNNNNgcata | |
| Tol2 nested F | gtacgagaatcgctgtcct | |
| **Genotyping** | | |
| γ1(R70Q) and *Prkag1* (-/-) genotyping | gaagacgtgttagggcttccg | cctgttcttgctccctccgctc |
| **CRISPR/CAS9 sgRNA and SSDNA** | | |
| sgRNA targeting *Prkag1* | ggtaagagcagcgcccctgtggg | |
| ssDNA donor | aaggcgttctttgctcttgtttctaacggggtacaagcagcgcccctttgggacagtaacaagcagtgctttg ttggtgagagcttgtgtcgtttcactgactttctgcataaa | |
