# Supplementary material for: Refeeding-associated AMPKγ1 complex activity is a hallmark of health and longevity
Source: Nat Aging. 2023 Nov 13;3(12):1544–60. doi: 10.1038/s43587-023-00521-y (PMC10724066; doi:10.1038/s43587-023-00521-y)
Supplement: Supplementary file 2 — Reporting Summary [file 43587_2023_521_MOESM2_ESM.pdf]

nature portfolio

Corresponding author(s): Adam Antebi

Last updated by author(s): 09-22.2023

Reporting Summary

Nature Portfolio wishes to improve the reproducibility of the work that we publish. This form provides structure for consistency and transparency in reporting. For further information on Nature Portfolio policies, see our Editorial Policies and the Editorial Policy Checklist.

Statistics

For all statistical analyses, confirm that the following items are present in the figure legend, table legend, main text, or Methods section.

n/a	Confirmed	
☐	☒	The exact sample size (*n*) for each experimental group/condition, given as a discrete number and unit of measurement
☐	☒	A statement on whether measurements were taken from distinct samples or whether the same sample was measured repeatedly
☐	☒	The statistical test(s) used AND whether they are one- or two-sided<br>*Only common tests should be described solely by name; describe more complex techniques in the Methods section.*
☐	☒	A description of all covariates tested
☐	☒	A description of any assumptions or corrections, such as tests of normality and adjustment for multiple comparisons
☐	☒	A full description of the statistical parameters including central tendency (e.g. means) or other basic estimates (e.g. regression coefficient) AND variation (e.g. standard deviation) or associated estimates of uncertainty (e.g. confidence intervals)
☐	☒	For null hypothesis testing, the test statistic (e.g. *F*, *t*, *r*) with confidence intervals, effect sizes, degrees of freedom and *P* value noted<br>*Give P values as exact values whenever suitable.*
☒	☐	For Bayesian analysis, information on the choice of priors and Markov chain Monte Carlo settings
☒	☐	For hierarchical and complex designs, identification of the appropriate level for tests and full reporting of outcomes
☐	☒	Estimates of effect sizes (e.g. Cohen's *d*, Pearson's *r*), indicating how they were calculated

Our web collection on statistics for biologists contains articles on many of the points above.

Software and code

Policy information about availability of computer code

Data collection	No code for data collection was generated in this study.<br>Here follows the list of the commercially available softwares used for data collection:<br>Microscopy: Leica application suite X 3.5.7.23225:<br>Gel DNA immaging and western blots: BioRad-ChemiDoc MP, Image lab 6.1
Data analysis	No code for data analysis was generated in this study<br>Here follows the list of the commercially available softwares used for data collection:<br>Statistical analysis: Graphpad Prism (9.0.0), Flaski (https://flaski.age.mpg.de/)<br>Histological quantifications: Imagej/Fiji (version 2.0/1.52p), Adiposoft (1.16)<br>Transcriptomic analysis: Kallisto (0.45.0), DESeq2 (1.24.0), ShinyGO (0.76.2), Flaski (https://flaski.age.mpg.de/)<br>MicroCT scan analysis: NRrecon software (Bruker), CTAn software (Bruker)

For manuscripts utilizing custom algorithms or software that are central to the research but not yet described in published literature, software must be made available to editors and reviewers. We strongly encourage code deposition in a community repository (e.g. GitHub). See the Nature Portfolio guidelines for submitting code & software for further information.

Data

Policy information about availability of data

All manuscripts must include a data availability statement. This statement should provide the following information, where applicable:

- Accession codes, unique identifiers, or web links for publicly available datasets
- A description of any restrictions on data availability
- For clinical datasets or third party data, please ensure that the statement adheres to our policy

There are no restrictions on data availability. Source data are provided with this paper. RNA-seq data can be found in the SRA database, bioProject ID: PRJNA817434.

Human research participants

Policy information about studies involving human research participants and Sex and Gender in Research.

Reporting on sex and gender	The human analyses were carried out both on males and females
Population characteristics	All the recruited participants were ≥ 65 years of age.
Recruitment	All internal medicine patients ≥ 65 years of age presenting to the Emergency Department of the University Hospital Cologne on a weekday between 9:00 am to 4:00 p.m. were screened consecutively by medically trained specialists with geriatric training. In principle, all patients who meet these criteria and can provide written declaration of consent can be included unless any of the following exclusion criteria are met. Exclusion criteria are persons unable to provide informed consent, persons who are in a dependency / employment relationship with the auditors, persons who are a placed in an institution based on a judicial or administrative order and persons who are unable to communicate in German. Patients who are unable to provide consent due to decreased general condition at the time of presentation to the ER can still be enrolled if they are admitted to the hospital and are able to provide consent within 7 days of initial presentation.<br>Thus, there are mainly two potential selection biases. Firstly, the group of people who are generally unwilling to participate in clinical trials will be missed. Secondly, extremely sick patients who remain unable to provide consent within 7 days of initial presentation will be missed. Both of these biases might be relevant when considering treatment interventions as part of the clinical trial. However, they are most likely irrelevant to this study as we solely correlate biomolecular characteristics with physical frailty but do not evaluate any kind of intervention.
Ethics oversight	Approval was obtained from the institutional review board of the University of Cologne (EK20-1346, EK19-1275), and written informed consent was obtained from all patients.

Note that full information on the approval of the study protocol must also be provided in the manuscript.

Field-specific reporting

Please select the one below that is the best fit for your research. If you are not sure, read the appropriate sections before making your selection.

☒ Life sciences ☐ Behavioural & social sciences ☐ Ecological, evolutionary & environmental sciences

For a reference copy of the document with all sections, see nature.com/documents/nr-reporting-summary-flat.pdf

Life sciences study design

All studies must disclose on these points even when the disclosure is negative.

Sample size

The exact sample size can be found in the figure legends. No statistical methods were used to calculate sample sizes, however our sample sizes are consistent with those reported in previous publications on the same topic:

Astre G, Atlan T, Goshtchevsky U, Oron-Gottesman A, Smirnov M, Shapira K, Velan A, Deelen J, Levy T, Levanon EY, Harel I. Genetic perturbation of AMP biosynthesis extends lifespan and restores metabolic health in a naturally short-lived vertebrate. Dev Cell. 2023 Aug 7;58(15):1350-1364.e10. doi: 10.1016/j.devcel.2023.05.015. Epub 2023 Jun 14. PMID: 37321215.

Pollard AE, Martins L, Muckett PJ, Khadayate S, Bornot A, Clausen M, Admyre T, Bjursell M, Fiadeiro R, Wilson L, Whilding C, Kotiadis VN, Duchen MR, Sutton D, Penfold L, Sardini A, Bohlooly-Y M, Smith DM, Read JA, Snowden MA, Woods A, Carling D. AMPK activation protects against diet induced obesity through Ucp1-independent thermogenesis in subcutaneous white adipose tissue. Nat Metab. 2019 Mar;1(3):340-349. doi: 10.1038/s42255-019-0036-9. Epub 2019 Feb 25. PMID: 30887000; PMCID: PMC6420092.

Yavari A, Stocker CJ, Ghaffari S, Wargent ET, Steeples V, Czibik G, Pinter K, Bellahcene M, Woods A, Martínez de Morentin PB, Cansell C, Lam BY, Chuster A, Petkevicius K, Nguyen-Tu MS, Martinez-Sanchez A, Pullen TJ, Oliver PL, Stockenhuber A, Nguyen C, Lazdam M, O'Dowd JF,

Harikumar P, Tóth M, Beall C, Kyriakou T, Parnis J, Sarma D, Katritsis G, Wortmann DD, Harper AR, Brown LA, Willows R, Gandra S, Poncio V, de Oliveira Figueiredo MJ, Qi NR, Peirson SN, McCrimmon RJ, Gereben B, Tretter L, Fekete C, Redwood C, Yeo GS, Heisler LK, Rutter GA, Smith MA, Withers DJ, Carling D, Sternick EB, Arch JR, Cawthorne MA, Watkins H, Ashrafian H. Chronic Activation of γ2 AMPK Induces Obesity and Reduces β Cell Function. Cell Metab. 2016 May 10;23(5):821-36. doi: 10.1016/j.cmet.2016.04.003. Epub 2016 Apr 28. PMID: 27133129; PMCID: PMC4873618.

Hartmann N, Reichwald K, Wittig I, Dröse S, Schmeisser S, Lück C, Hahn C, Graf M, Gausmann U, Terzibasi E, Cellerino A, Ristow M, Brandt U, Platzer M, Englert C. Mitochondrial DNA copy number and function decrease with age in the short-lived fish Nothobranchius furzeri. Aging Cell. 2011 Oct;10(5):824-31. doi: 10.1111/j.1474-9726.2011.00723.x. Epub 2011 Jun 27. PMID: 21624037.

Martin-Montalvo A, Mercken EM, Mitchell SJ, Palacios HH, Mote PL, Scheibye-Knudsen M, Gomes AP, Ward TM, Minor RK, Blouin MJ, Schwab M, Pollak M, Zhang Y, Yu Y, Becker KG, Bohr VA, Ingram DK, Sinclair DA, Wolf NS, Spindler SR, Bernier M, de Cabo R. Metformin improves healthspan and lifespan in mice. Nat Commun. 2013;4:2192. doi: 10.1038/ncomms3192. PMID: 23900241; PMCID: PMC3736576.

Data exclusions

No data were excluded from our analysis

Replication

The precise number of biological replicates, which corresponds to the number of individual fish or human samples used for each experiment in the study, is consistently provided in the figure legends.
All fish experiments were replicated at least twice using an independent cohort of fish to rule out batch effects
Transcriptomic data were generated through a single experiment but were consistently validated using qPCR on an independent cohort of fish.

Randomization

Fish were indiscriminately allocated to groups for all experiments in this study.

Blinding

Histological and survival analyses were conducted blindly.

Reporting for specific materials, systems and methods

We require information from authors about some types of materials, experimental systems and methods used in many studies. Here, indicate whether each material, system or method listed is relevant to your study. If you are not sure if a list item applies to your research, read the appropriate section before selecting a response.

Materials & experimental systems

n/a	Involved in the study
☐	☒ Antibodies
☒	☐ Eukaryotic cell lines
☒	☐ Palaeontology and archaeology
☐	☒ Animals and other organisms
☐	☒ Clinical data
☒	☐ Dual use research of concern

Methods

n/a	Involved in the study
☒	☐ ChIP-seq
☒	☐ Flow cytometry
☒	☐ MRI-based neuroimaging

Antibodies

Antibodies used

Rabbit anti-total AMPKα (CST:2532; 1:1000)
Rabbit anti-phospho(Thr172)-AMPKα (CST:2535; 1:1000)
Rabbit anti-total AMPKβ (CST:4150;1:1000)
Rabbit anti-total-ACC (CST:3676; 1:500)
Rabbit anti-phospho(Ser79)-ACC (CST:3661; 1:1000)
Rabbit anti-γ2 (Invitrogen: PA522331; 1:1000)
Mouse anti-alpha-tubulin (Sigma:T6074; 1:10000)
Anti-mouse HRP (Thermofisher: G-21040; 1:5000)
Anti-rabbit HRP (Thermofisher: G-21234; 1:5000)
Rabbit anti-L-plastin (Genetex: GTX124420; 1:400)
Total-ribosomal S6 (CST:2317, 1:000)
Phospho(ser235) S6 (CST:2211, 1:1000)
OXPHOS cocktail (Abcam:ab110413;1;1000)

Validation

All the antibodies related to the AMPK subunits components were validated in this study using the AMPK gamma1 K.O line:

Previous studies in mice have demonstrated that genetic ablation of the gamma1 subunit results in a significant reduction of the beta and alpha subunits, while the gamma2 subunit remains largely unaffected:

Foretz M, Hébrard S, Guihard S, Leclerc J, Do Cruzeiro M, Hamard G, Niedergang F, Gaudry M, Viollet B. The AMPKγ1 subunit plays an essential role in erythrocyte membrane elasticity, and its genetic inactivation induces splenomegaly and anemia. FASEB J. 2011 Jan;25(1):337-47. doi: 10.1096/fj.10-169383. Epub 2010 Sep 29. PMID: 20881209.

An H, Wang Y, Qin C, Li M, Maheshwari A, He L. The importance of the AMPK gamma 1 subunit in metformin suppression of liver glucose production. Sci Rep. 2020 Jun 26;10(1):10482. doi: 10.1038/s41598-020-67030-5. PMID: 32591547; PMCID: PMC7320014.

Consistently, we established a gamma1-/- killifish line, and through Western blot analysis, we observed a substantial decrease in the alpha and beta subunits, while the gamma2 subunit remained unaffected. Notably, all protein bands exhibited the expected molecular weights, in line with the in silico predictions for this species.

P-ACC and Total ACC , P-S6 nad total P-S6 antibodies were previously validated in:

Astre G, Atlan T, Goshtchevsky U, Oron-Gottesman A, Smirnov M, Shapira K, Velan A, Deelen J, Levy T, Levanon EY, Harel I. Genetic perturbation of AMP biosynthesis extends lifespan and restores metabolic health in a naturally short-lived vertebrate. Dev Cell. 2023 Aug 7;58(15):1350-1364.e10. doi: 10.1016/j.devcel.2023.05.015. Epub 2023 Jun 14. PMID: 37321215.

L-plastin antibody was validated in:

Van Houcke J, Mariën V, Zandecki C, Vanhunsel S, Moons L, Ayana R, Seuntjens E, Arckens L. Aging impairs the essential contributions of non-glial progenitors to neurorepair in the dorsal telencephalon of the Killifish Nothobranchius furzeri. Aging Cell. 2021 Sep;20(9):e13464. doi: 10.1111/acel.13464. Epub 2021 Aug 24. PMID: 34428340; PMCID: PMC8441397.

Animals and other research organisms

Policy information about studies involving animals; ARRIVE guidelines recommended for reporting animal research, and Sex and Gender in Research

Laboratory animals	Nothobranchius furzeri wild type strain: GRZ-AD, genders: both, age: 7-8 weeks (young adults), 18-20 weeks (old)<br>Nothobranchius furzeri Ubi:gamma1(R70Q), genders: both, age: 7-8 weeks (young adults), 18-20 weeks (old)<br>Nothobranchius furzeri Gamma1(R70Q), genders: both, age: 7-8 weeks (young adults), 18-20 weeks (old)<br>Nothobranchius furzeri Prkag1-/-, genders: males, age: 7-8 weeks (young adults)
Wild animals	No wild animals were used in the study
Reporting on sex	All the experiments were conducted on males only except for survival analyses and fat measurements, where bot sexes were used.
Field-collected samples	No filed collected samples were used in the study
Ethics oversight	Animal experimentation was approved by "Landesamt für Natur, Umwelt und Verbraucherschutz Nordrhein-Westfalen": 81-02.04.2019.A055.

Note that full information on the approval of the study protocol must also be provided in the manuscript.

Clinical data

Policy information about clinical studies

All manuscripts should comply with the ICMJE guidelines for publication of clinical research and a completed CONSORT checklist must be included with all submissions.

Clinical trial registration	German Clinical studies Register DRKS00017365 and DRKS00024592
Study protocol	Detailed study protocol can be found at https://drks.de/search/download/attachment;jsessionid=273419FA283DF84D4353AE8522E45A29?reference=ba3f4e59-110e-4095-99b4-a4a4813dded9
Data collection	Monocentric study at University Hospital Cologne, Germany. Start of study: 01.06.2020. Planned number of participants: 1000. No predetermined end date of the study. The study will be completed once sufficient participants have been included.
Outcomes	The human biomaterial analysed for this study was obtained from patients participating in both of the above-mentioned clinical studies. DRKS00017365 examines the prospective outcome of elderly patients presenting to the emergency room in correlation with frailty assessments. The primary and secondary endpoints of this study, outlined below, are explained in detail in the trial registration entry in the German Clinical Trials Register (DRKS00017365). However, these endpoints are not part of the manuscript at hand. The manuscript at hand primarily describes results of the second study, DRKS00024592, which provides biosamples and uses them to elucidate biomolecular characteristics of ageing (as described in the respective entry in the German Clinical Trials Register). The endpoint of DRKS00024592 is exploratory in nature (biomolecular characterization of aging in human biosamples).<br>However, DRKS00017365 is also mentioned since – in the manuscript at hand - the biomolecular results are correlated with the multiparametric prognostic index (MPI), obtained in DRKS00024592. The assessment of clinical characteristics used for the correlation analysis – namely the multidimensional prognostic index (MPI) – is described in depth in the methods section of this study.<br><br>Primary endpoint: Rehospitalization days after 12 months as part of a telephone follow-up<br>Secondary endpoint:<br>1. rehospitalization, reason for rehospitalization<br>2. mortality

3. implementation of geriatric recommendations in further treatment
4. VAS quality of life (EQ-5D-5L)
5. geriatric depression scale (GDS)
6. number of precautionary measures (health care proxy, living will)
7. degree of care
8. use of care service provision
9. use of nursing home accommodation
10. number of medications
11. number of falls
12. nutrient ratio of the diet and food group balance (through Freiburg Nutrition Protocol)
13. multidimensional prognostic index (MPI)
