# Supplementary figures and images for: Refeeding-associated AMPKγ1 complex activity is a hallmark of health and longevity
Source: Nat Aging. 2023 Nov 13;3(12):1544–60. doi: 10.1038/s43587-023-00521-y (PMC10724066; doi:10.1038/s43587-023-00521-y)

Figure 3F

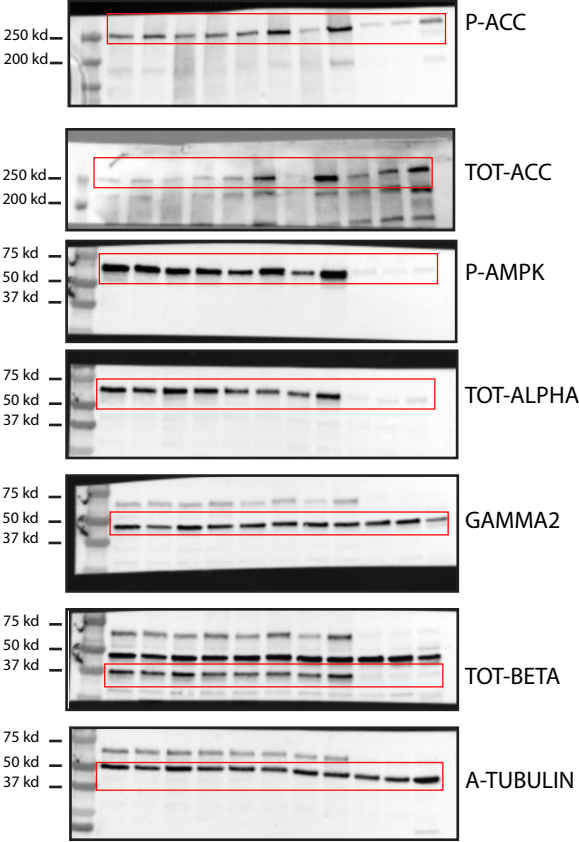

Figure 3H

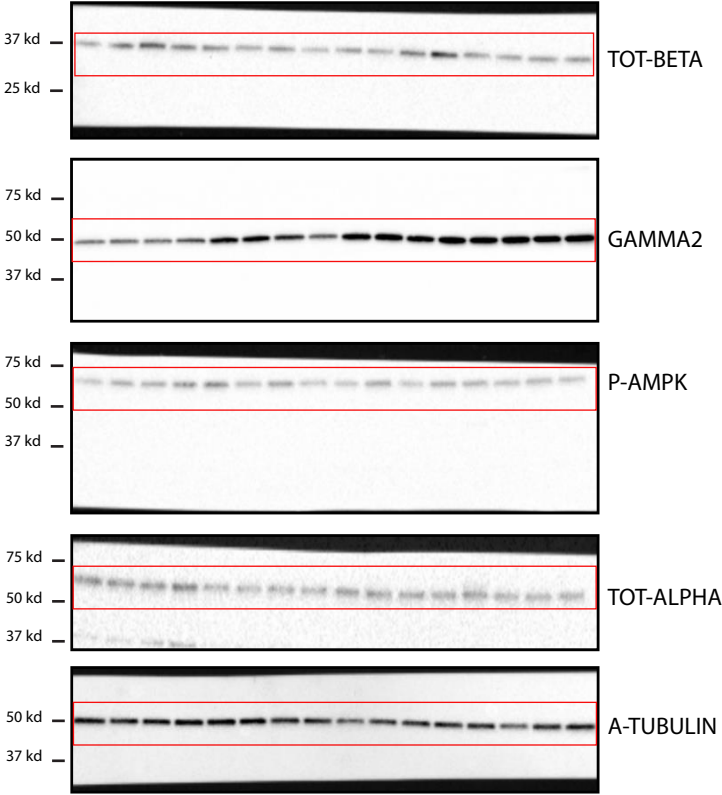

Supplement: Supplementary file 6 — Fig. 3 Unprocessed western blots. [file 43587_2023_521_MOESM6_ESM.pdf]

Extended data Figure 2g

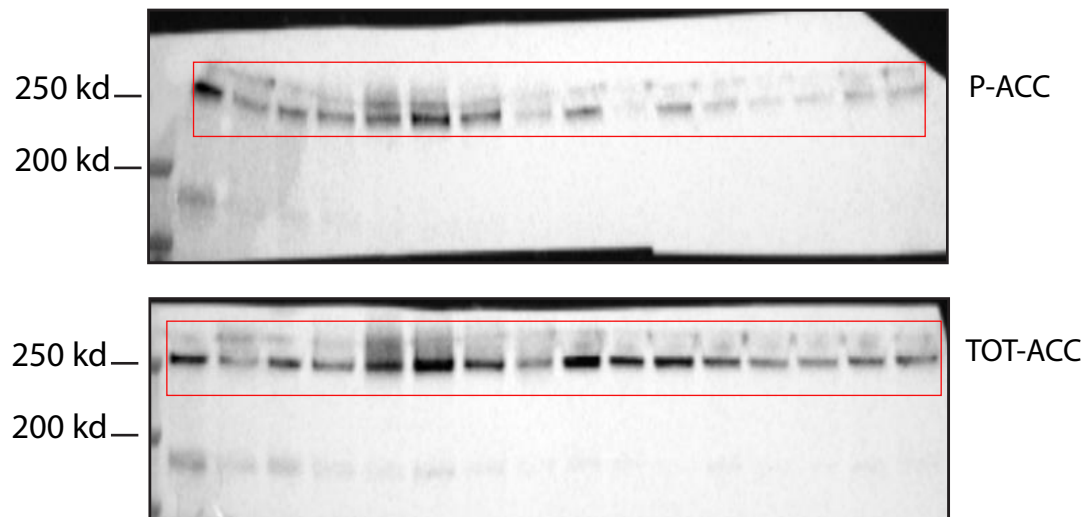

Extended data Figure 2i

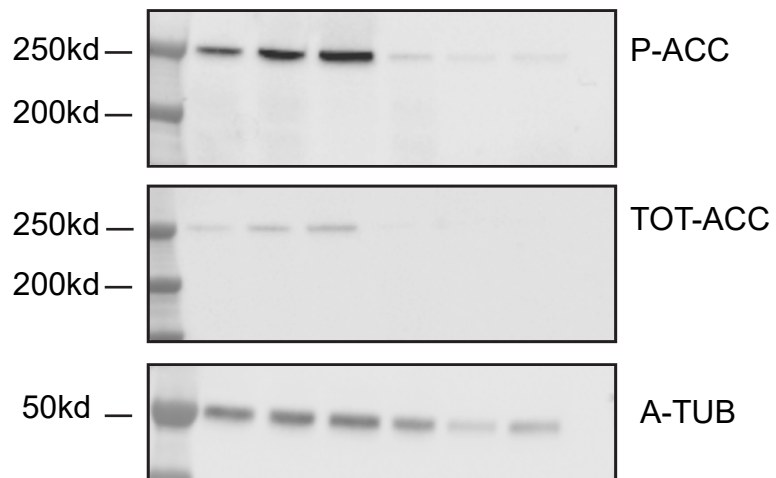

Supplement: Supplementary file 12 — Extended Data Fig. 2 Unprocessed western blots. [file 43587_2023_521_MOESM12_ESM.pdf]

Extended data figure 3C

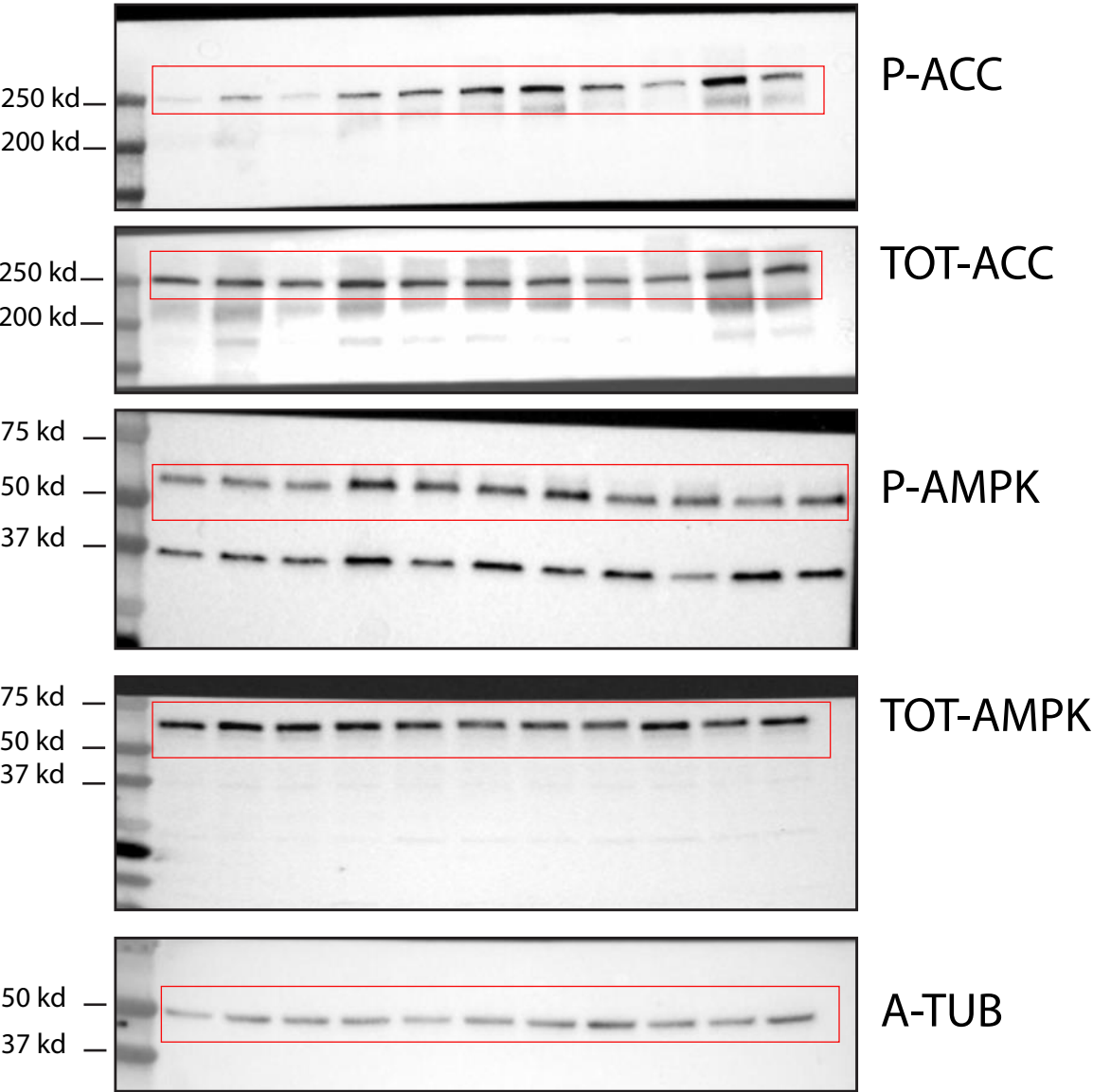

Supplement: Supplementary file 14 — Extended Data Fig. 3 Unprocessed western blots. [file 43587_2023_521_MOESM14_ESM.pdf]

Extended data figure 4c

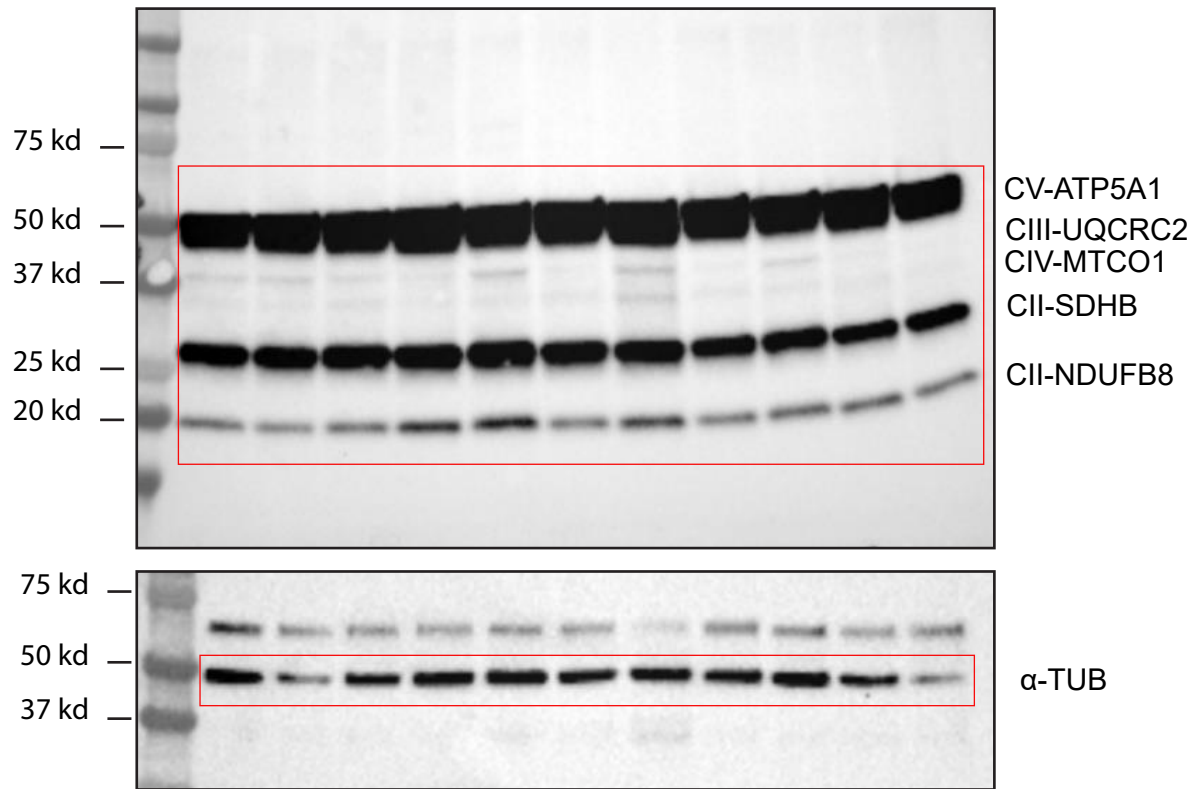

Supplement: Supplementary file 16 — Extended Data Fig. 4 Unprocessed western blots. [file 43587_2023_521_MOESM16_ESM.pdf]

Extended data figure 5F

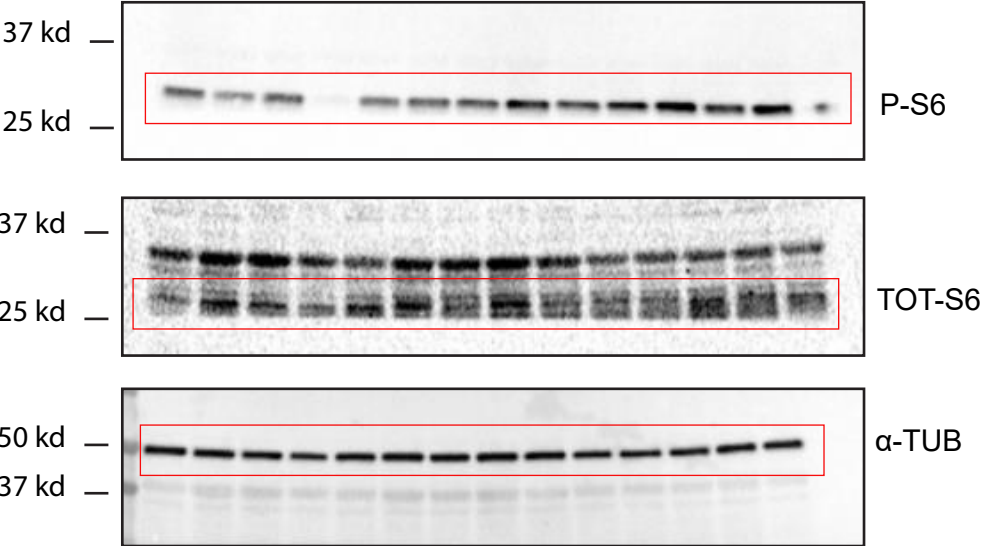

Supplement: Supplementary file 19 — Extended Data Fig. 6 Unprocessed western blots. [file 43587_2023_521_MOESM19_ESM.pdf]
